# Temporal dynamics of genetic clines of invasive European green crab (*Carcinus maenas*) in eastern North America

**DOI:** 10.1111/eva.12657

**Published:** 2018-06-28

**Authors:** Sarah J. Lehnert, Claudio DiBacco, Nicholas W. Jeffery, April M. H. Blakeslee, Jonatan Isaksson, Joe Roman, Brendan F. Wringe, Ryan R. E. Stanley, Kyle Matheson, Cynthia H. McKenzie, Lorraine C. Hamilton, Ian R. Bradbury

**Affiliations:** ^1^ Northwest Atlantic Fisheries Centre Fisheries and Oceans Canada St. John's Newfoundland Canada; ^2^ Bedford Institute of Oceanography Fisheries and Oceans Canada Dartmouth Nova Scotia Canada; ^3^ Biology Department East Carolina University Greenville North Carolina; ^4^ Gund Institute for Environment University of Vermont Burlington Vermont; ^5^ Aquatic Biotechnology Laboratory Bedford Institute of Oceanography Dartmouth Nova Scotia Canada

**Keywords:** aquatic invasive species, genetic cline, hybrid zone, secondary contact, single nucleotide polymorphisms

## Abstract

Two genetically distinct lineages of European green crabs (*Carcinus maenas*) were independently introduced to eastern North America, the first in the early 19th century and the second in the late 20th century. These lineages first came into secondary contact in southeastern Nova Scotia, Canada (NS), where they hybridized, producing latitudinal genetic clines. Previous studies have documented a persistent southward shift in the clines of different marker types, consistent with existing dispersal and recruitment pathways. We evaluated current clinal structure by quantifying the distribution of lineages and fine‐scale hybridization patterns across the eastern North American range (25 locations, ~39 to 49°N) using informative single nucleotide polymorphisms (SNPs; *n* = 96). In addition, temporal changes in the genetic clines were evaluated using mitochondrial DNA and microsatellite loci (*n* = 9–11) over a 15‐year period (2000–2015). Clinal structure was consistent with prior work demonstrating the existence of both northern and southern lineages with a hybrid zone occurring between southern New Brunswick (NB) and southern NS. Extensive later generation hybrids were detected in this region and in southeastern Newfoundland. Temporal genetic analysis confirmed the southward progression of clines over time; however, the rate of this progression was slower than predicted by forecasting models, and current clines for all marker types deviated significantly from these predictions. Our results suggest that neutral and selective processes contribute to cline dynamics, and ultimately, highlight how selection, hybridization, and dispersal can collectively influence invasion success.

## 1 INTRODUCTION

Marine genetic studies increasingly demonstrate patterns of population structure over much smaller scales than might be predicted simply by dispersal potential (Sa‐Pinto, Baird, Pinho, Alexandrino, & Branco, 2010; Selkoe, Henzler, & Gaines, 2008; Selkoe et al., 2016; Väinölä & Hvilsom, 1991), including the existence of genetic clines across tens to hundreds of kilometres (Bradbury et al., 2010; Hare & Avise, 2011; Pringle, Blakeslee, Byers, & Roman, 2011; Sotka, Wares, Barth, Grosberg, & Palumbi, 2004). Genetic clines can arise via secondary contact between previously isolated populations and/or natural selection along an environmental gradient, where the formation of transitional (or hybrid) zones may exist as stable or moving boundaries between genetically distinct individuals (Barton & Hewitt, 1985; Nagylaki, 1975). Recent marine genomic studies have observed clinal patterns in the native range of species such as Atlantic cod *Gadus morhua* (Bradbury et al., 2010), European anchovy *Engraulis encrasicolus* (Silva, Lima, Martel, & Castilho, 2014), American lobster *Homarus americanus* (Benestan et al., 2016) and sea scallop *Placopecten magellanicus* (Van Wyngaarden et al., 2017). However, determining the mechanisms ultimately responsible for genetic clines can be challenging, as structure can be a product of both adaptation and historical vicariance.

Species invasions therefore provide a unique opportunity to investigate clines formed by contemporary interactions and observe these evolutionary processes in action. Several marine invasive species are characterized by genetic structure in their invaded range that can arise through different mechanisms such as multiple divergent source introductions, hybridization, and/or range expansion with subsequent divergence (Herborg, Weetman, Van Oosterhout, & Hänfling, 2007; Pringle et al., 2011; Richardson, Sherman, Lee, Bott, & Hirst, 2016; Roman & Darling, 2007; Saarman & Pogson, 2015). For example, in blue mussels (genus *Mytilus*), genetic clines have been formed through contact and introgression between invasive (*M. galloprovincialis*) and native (*M. trossulus*) mussels in the Pacific Northwest (Saarman & Pogson, 2015). Genetic clines have also been formed when invasion fronts from two separate introductions from previously allopatric lineages come into secondary contact in the invaded range, and such a scenario has occurred in the invasive European green crab (*Carcinus maenas*) (Darling, Tsai, Blakeslee, & Roman, 2014; Pringle et al., 2011; Roman, 2006).

*Carcinus maenas* is native to Europe and northern Africa and is among the most notorious marine invasive species worldwide (Darling, Bagley, Roman, Tepolt, & Geller, 2008; Roman, 2006). *C. maenas* have invaded the waters of multiple continents including the Atlantic and Pacific coasts of North America (Darling et al., 2008; Roman, 2006), and their introductions can have significant impacts on invaded ecosystems, including damage to eelgrass beds (Garbary, Miller, Williams, & Seymour, 2013; Malyshev & Quijon, 2011), agonistic and predatory interactions with native species (Floyd & Williams, 2004; Rossong, Williams, Comeau, Mitchell, & Apaloo, 2006) and overall alterations to community structure (Lutz‐Collins, Cox, & Quijón, 2016; Matheson et al., 2016). Along the coast of eastern North America, molecular data support that the current distribution of *C. maenas* is the result of at least two separate introductions. The initial introduction was reported in 1817 near New York, United States (Say, 1817) and likely originated from southwestern Europe (Roman, 2006). Following the initial US introduction, *C. maenas* expanded northward to Nova Scotia, Canada (NS) during the 1900s, but further movement appeared to halt along the eastern Scotian Shelf near Halifax, NS (~44.6°N) (Roman, 2006). A second genetically distinct introduction originating from northern Europe likely occurred in the 1980s in northeastern NS and the Gulf of St. Lawrence (Blakeslee et al., 2010; Darling et al., 2008; Roman, 2006). Over the last two decades, this northern lineage has subsequently spread through NS and New Brunswick (NB) and has made secondary contact with the original southern introduction. As a result, hybridization between the lineages has occurred in these areas resulting in latitudinal genetic clines (Darling et al., 2014; Jeffery et al., 2017a; Pringle et al., 2011). In addition, anthropogenic transport of individuals from the zone of secondary contact has resulted in a recent introduction of admixed individuals in southeastern Newfoundland (NL) (Blakeslee et al., 2010).

In this system, we take advantage of the strong genetic differentiation between lineages from the past and recent introductions, and the interaction of invasion fronts, as a platform to examine dynamics in the hybrid zone and study the temporal changes in the genetic clines. Such investigations are possible because the clinal genetic structure of *C. maenas* in eastern North America has been well characterized in previous studies (Darling et al., 2014; Jeffery et al., 2017a; Pringle et al., 2011), allowing for a historical perspective and thus the ability to investigate temporal dynamics. Previous studies exploring *C. maenas* genetic structure between 1999 and 2007 documented a southward progression of the genetic clines (Darling et al., 2014; Pringle et al., 2011), consistent with the predominant southward circulation on the eastern Scotian Shelf (DFO, 2003; Wu, Tang, & Hannah, 2012) and the broad dispersal potential of larval *C. maenas*, which can remain in the water column for >50 days (Behrens Yamada et al., 2005; Klassen & Locke, 2007; Pringle et al., 2011). Considering the temporal movement of the clines, it was predicted that continued patterns of dispersal and connectivity would result in a persistent southward shift (Pringle et al., 2011). Darling et al. (2014), however, suggested that larval dispersal alone could not explain the displacement of *C. maenas* clines, where introgression occurred more rapidly for mitochondrial DNA relative to nuclear markers, suggesting demographic processes were influencing invasion dynamics. Indeed, predictions made by Pringle et al. (2011) do not explicitly account for demographic processes such as gene surfing (Slatkin & Excoffier, 2012) or the role of hybridization in countering Allee effects (Mesgaran et al., 2016), nor do they account for selection associated with potential adaptive differences among invasions (Jeffery et al., 2018; Tepolt & Somero, 2014). While demographic processes have been implicated for changes in *C. maenas* genetic clines, recent evidence has suggested there might be differences in physiological thermal tolerance and behaviour between crabs from different regions (Rossong et al., 2011; Tepolt & Palumbi, 2015; Tepolt & Somero, 2014) that could influence invasion and range expansion success (Williams, Nivison, Ambrose, Dobbin, & Locke, 2015).

Here, we examine the distribution patterns of *C. maenas* lineages in eastern North America from New Jersey, United States, to western NL, Canada (~39 to 49°N) between 2011 and 2015 and compare to previous studies. We used single nucleotide polymorphisms (SNPs) selected to discriminate between northern and southern lineages, building directly on previous studies of population structure using SNPs (Jeffery et al., 2017a, 2017b) by expanding the scale of sampling, both in number of samples and by life stage (juveniles and adults). In addition, to evaluate the temporal progression of the clines, we incorporated genetic data collected over several time points from 2000 to 2015 from both mitochondrial and microsatellite markers. With these samples, we quantified the temporal changes in cline position and shape across different marker types, thus providing a new perspective into the evolutionary processes that shape the current clines. This work represents the most comprehensive spatial genetic census using multiple marker types in the *C. maenas* invasive western Atlantic range to date. Our ability to determine the current distribution and movement patterns of lineages over the last decade is critical to the ongoing management and monitoring of this invasive species as lineages could have differential impacts on ecosystems given differences found in physiology, reproduction and competitive behaviour of crabs from different regions (Best, McKenzie, & Couturier, 2017; Rossong et al., 2011; Tepolt & Palumbi, 2015; Tepolt & Somero, 2014).

## 2 METHODS

### 2.1 Sample collection and DNA extraction for SNP genotyping

Adult crabs were collected from 25 sites along the coast of eastern North America between 2011 and 2015 (Table 1). Juvenile crabs were also collected from a subset of 15 sites in 2015 to quantify genetic differences between life stages, to obtain a current understanding of the species’ population structure and clinal patterns, and potentially detect natural selection acting as one mechanism regulating the spread of lineages. The use of 15 sampling locations for both adults and juveniles also allowed us to compare changes in the genetic clines between life stages. All juvenile crabs were <25 mm in carapace width and likely represent juveniles that have already experienced one winter, as previous research found that juvenile crabs in Maine were 3–10 mm at the end of their first winter and 13–28 mm in carapace width after the second winter (Berrill, 1982). Our sampling design included many sites located within the zone of secondary contact where lineages have met and hybridized, allowing us to accurately estimate the current cline centre and examine fine‐scale patterns of hybridization in this region. Tissue samples were preserved in AllProtect (Qiagen, Toronto, ON, Canada) or 80% ethanol. DNA was isolated from the tissue samples using phenol:chloroform extraction or NucleoMag 96 Tissue kit (Macherey‐Nagel, Bethlehem, PA, USA) following the manufacturer's protocol, without optional RNase A (Qiagen) treatment. All DNA samples were quantified following the same protocol as Jeffery et al. (2017b) using Quant‐iT PicoGreen dsDNA Assay Kit (Thermo Fisher Scientific, Waltham, MA USA) and the FLUOStar OPTIMA fluorescence plate reader (BMG Labtech, Ortenberg, Germany).

**Table 1:** Sampling locations and code of European green crab (*Carcinus maenas*) with sampling year and the number of adults and juveniles genotyped at 96 high‐*F*_ST_ SNPs

Sampling location	Code	Latitude	Longitude	Year collected	Adults	Juveniles
Tuckerton, NJ^a^	TKT	39.588	−74.288	2011	22	
New Hampshire^a^	NWH	43.040	−70.710	2013	22	
Campobello Island, NB^a^	CBI	44.888	−66.918	2011	22	
St. Andrew's, NB	STA	45.069	−67.041	2015	32	32
Musquash, NB	MSQ	45.184	−66.247	2015	32	32
Two Rivers Inlet, NB	CMB	45.656	−64.725	2015	32	32
Hampton, NS	HMP	44.906	−65.352	2015	32	32
Gunning Cove, NS	GUN	43.679	−65.342	2015	32	31
Yarmouth Bar, NS	YRM	43.802	−66.152	2015	32	31
Kejimkujik National Park, NS^a^	KJI	43.840	−64.836	2011/2015	53	31
East River Point, NS	ERV	44.574	−64.158	2015	32	32
Cole Harbour, NS^a^	CLH	44.653	−63.425	2011/2015	54	32
Port Bickerton, NS	PTB	45.094	−61.729	2015	32	31
Sydney Harbour, NS^a^	SYH	46.141	−60.202	2011	22	
Mabou, NS^a^	MBO	46.069	−61.391	2011	22	
Brudenell River, PE^a^	BRN	46.192	−62.588	2011	22	
Bouctouche Head, NB	BCT	46.500	−64.677	2015	32	
Baie de Bassin, QC^a^	BDB	47.473	−61.738	2011	22	
Fortune Bay, NL (Little Harbour East)	FTB	47.593	−54.859	2015	32	32
Boat Harbour, NL	BTH	47.431	−54.837	2015	32	30
North Harbour, NL^a^	NOH	47.855	−54.098	2011/2015	54	32
Fair Haven, NL	FRH	47.539	−53.893	2015	32	28
St. George's Bay, NL^a^^,^ b	SGB	48.492	−58.658	2011	22	
Port Harmon, NL^b^	PTH	48.525	−58.537	2015	32	31
Corner Brook, NL	CNB	48.959	−57.987	2015	32	

*Notes*.

Juvenile crabs were collected from a subset of locations in 2015.

a Indicates that some or all adult samples were previously genotyped by Jeffery et al. (2017b).

b We note that these sites are located in close proximity but considered separately here.

### 2.2 SNP genotyping

A total of 9,137 single nucleotide polymorphisms (SNPs) were previously genotyped using restriction site‐associated DNA sequencing (RAD‐seq) on *C. maenas* collected in 2011 and 2013 (see Jeffery et al., 2017b). Locus‐specific *F*_ST_ values were calculated for all SNPs across all *C. maenas* populations from Jeffery et al. (2017b), and using these values, we chose a panel of collectively informative SNPs for differentiating between lineages, which included SNPs with the highest *F*_ST_ and low linkage disequilibrium (Supporting Information Table S1 and Supporting Information Figure S1). Candidate SNP Type assays (Fluidigm, San Francisco, CA, USA) were tested on a set of samples including those used to generate the RAD‐seq libraries because these would have known genotypes at the target loci (Jeffery et al., 2017b). Loci were selected for inclusion in the final panel of SNPs based on the ranking of the target SNP in the prioritized list as well as the results of the SNP Type assay, where we required that the locus had (a) a cluster pattern that was easy to interpret, (b) correct genotypes for known samples and positive controls (see below) and (c) genotypes that were reproducible across multiple chip runs. The final panel consisted of 96 SNPs (see Supporting Information Table S1). Although loci were not fixed between north and south, these SNPs were capable of discriminating between the northern and southern lineage, as the mean pairwise *F*_ST_ value between the southernmost and northernmost site was 0.344 (range 0.071–0.802) (see Supporting Information Figure S1B). gBlocks (Integrated DNA Technologies, Coralville, IA, USA) were designed and synthesized for use as positive controls (Richards‐Hrdlicka, 2014).

All crabs collected in 2015 were genotyped at the selected 96 SNP panel using SNP Type assays (Fluidigm) following the manufacturer's protocols, including the STA (Specific Target Amplification) step, using 96.96 genotyping Integrated Fluidic Circuits (IFC) and read on an EP1 platform (Fluidigm) and analysed using SNP Genotyping Analysis software (Fluidigm). Each 96‐well extraction included 10 samples that were repeated on the plate (i.e., redundants) to ensure there were no processing errors (row or plate reversal) and to ensure consistent clustering interpretation. The set‐up for each IFC also included gBlocks as positive controls (see above for details). After genotyping analysis, individual samples below the genotype quality threshold (>9 failed loci) were removed from the data set (6 samples in total). A subset of samples (12%) was reanalysed, from the original tissue where permitted, to calculate the genotype error rate. The genotype error rate was calculated to be 0.16% based on methods outlined in Pompanon, Bonin, Bellemain, and Taberlet (2005). This is consistent with the range (0%–0.2%) found in previous studies (Hess et al., 2015; Larson et al., 2013; Petrou et al., 2013). After this quality checking and prior to genetic analyses, genotypes generated from the SNP assays were merged with genotypes for the 96 SNPs from the previous RAD‐seq data set using the R package *genepopedit* (Stanley, Jeffery, Wringe, DiBacco, & Bradbury, 2017).

### 2.3 Population structure

First, to test for genetic differences between life stages in our SNP data, we conducted comparisons for juvenile and adult samples collected from the same location. ARLEQUIN v3.5 (Excoffier & Lischer, 2010) was used to calculate pairwise genetic divergence (*F*_ST_) between all adult and juvenile samples collected in 2015. *p*‐values were corrected to control for false discovery rate (FDR) using the p.adjust function in R software (R Core Development Team, 2016). The genetic distances among life stages and site locations for our 2015 samples were also compared using a neighbour‐joining tree based on Cavalli‐Sforza and Edwards (1967) chord distances calculated in POPULATIONS v1.2.33 (Langella, 2012) with 1,000 bootstrap replicates. FigTree v1.4 (Rambaut, 2012) was used to visualize the genetic relationships. Additional site‐specific comparisons for our SNP data set included differences between sampling years for the three sites in our study that were sampled in both 2011 and 2015. Pairwise *F*_ST_ values were calculated between adults collected 4 years apart from Kejimkujik, NS (KJI), Cole Harbour, NS (CLH) and North Harbour, NL (NOH). Differences between the genetic clines between sampling year and life stages were also investigated (see below and Supporting Information). The overall results of these comparisons across life stages and sampling year for our SNP data set guided all subsequent analyses of our study. Because comparisons provided little evidence of genetic differences between 2011 and 2015 as well as between life stages (Table 2; see also Section 3 and Supporting Information Figures S2, S3, S4, and Supporting Information Table S2), we pooled all samples for subsequent analyses of SNP data.

**Table 2:** Pairwise *F*_ST_ values with corresponding significance (*p*‐values corrected for false discovery rate, FDR) for European green crab (*Carcinus maenas*) adults and juveniles collected in 2015 from the same sampling location

Location	Adult–Juvenile *F*_ST_	*p*‐value
STA	−0.0031	0.942
MSQ	0.0016	0.307
CMB	−0.0018	0.779
HMP	0.0175	0.003*
YRM	−0.0016	0.810
GUN	0.0019	0.314
KJI	0.0018	0.307
ERV	−0.0047	1.000
CLH	−0.0014	0.717
PTB	0.0063	0.053
FTB	0.0105	0.002*
BTH	−0.0013	0.768
NOH	0.0047	0.636
FRH	−0.0010	0.065
PTH	−0.0003	0.545

*Note*.

Significant pairwise comparisons are indicated by an asterisk (*) after FDR correction.

Spatial genetic structuring across the sampling range was determined using Bayesian clustering analysis in the program STRUCTURE v2.3.4 (Pritchard, Stephens, & Donnelly, 2000). STRUCTURE runs were completed through the R package *parallelstructure* (Besnier & Glover, 2013) to perform three independent Markov Chain Monte Carlo (MCMC) runs using 100,000 burn‐in and 500,000 iterations for *K* = 2 (i.e., two genetic clusters). We chose *K* = 2 because we expected to identify two genetic clusters (derived from northern and southern lineages) with hybridization occurring in parts of the range based on previous *C. maenas* studies, their known invasion history and the informative SNPs chosen for our study (Blakeslee et al., 2010; Darling et al., 2008; Jeffery et al., 2017a; Pringle et al., 2011; Roman, 2006). In addition, higher *K* values in STRUCTURE revealed little sign of further substructuring. CLUMPAK (Kopelman, Mayzel, Jakobsson, Rosenberg, & Mayrose, 2015) was used to visualize clusters and calculate the proportion of membership to each cluster for each individual and site across runs.

### 2.4 Hybrid assignment

To assign individuals as hybrids between the northern and southern lineage within our sample, we used the program NEWHYBRIDS v1.1 (Anderson, 2008). Following methods described for *C. maenas* hybrid assignment in Jeffery et al. (2017a), we tested the ability of our 96 SNPs to accurately identify hybrids and then assigned individuals to hybrid and pure classes in our data set (see Supporting Information for full details of hybrid analyses). Hybrid assignments were evaluated at two levels, where our primary analysis included three genotype classes (pure north, pure south and hybrid). For the secondary analysis, we report hybrid classes as two groups: F_1_ hybrids and recombinant hybrids, with the latter class including F_2_, backcrosses and potentially later generation hybrids (e.g., F_3_ and later generations). We chose to consider only a single recombinant hybrid class because these later generation hybrids are unreliably detected. Next, we validated our hybrid results using GENODIVE (Meirmans & Van Tienderen, 2004) (see Supporting Information for full details).

Hybridization patterns in the hybrid zone and the introduced hybrid region in southeastern NL were further investigated using the R package *INTROGRESS* (Gompert & Buerkle, 2010). *INTROGRESS* was used to calculate maximum‐likelihood hybrid indices as well as interlineage heterozygosity to generate triangle plots and examine potential differences in levels of introgression (i.e., generation of hybridization) between the two hybrid regions. Simulated data generated from *hybriddetective* (Wringe, Stanley, Jeffery, Anderson, & Bradbury, 2017) (see Supporting Information) were used to distinguish between potential hybrid generations, including an additional third‐generation hybrid (F_3_) group (simulated F_2_ × F_2_ crosses). All individuals within southeastern NL (four sites) and the hybrid zone (including all sites between St. Andrews, NB to East River Point, NS) were used for *INTROGRESS* analyses with simulated pure north and pure south individuals used as the parental populations.

### 2.5 Temporal changes in the genetic clines

In addition to the informative SNPs described above, we also included two additional types of marker (mitochondrial and microsatellite loci) to provide further information about the processes shaping the genetic clines and to compare marker types, particularly because different marker types have been shown in *C. maenas* to differ in their population frequencies and clinal progression over time (Darling et al., 2014). In addition, these two marker types provide a historical understanding of changes in *C. maenas* population structure over time (Darling et al., 2014; Pringle et al., 2011; Roman, 2006). We modelled the genetic clines of *C. maenas* over multiple time points since 2000 using the mitochondrial cytochrome *c* oxidase subunit I (COI) gene and microsatellite markers (see Supporting Information for detailed methods of sampling and genotyping). In brief, genotypes for *C. maenas* collected in 2000, 2002 and 2007 were compiled from previous studies (Blakeslee et al., 2010; Darling et al., 2014; Pringle et al., 2011; Roman, 2006). Using the same methodology, in summer 2015, young‐of‐the‐year *C. maenas* were collected and genotyped at the COI and 11 microsatellite markers (see Supporting Information).

To evaluate the temporal changes to the clines as well as the current genetic clines of *C. maenas* in North America, we used the same approach as Darling et al. (2014), where maximum‐likelihood (ML) cline models were generated in the R package *hzar* (Derryberry, Derryberry, Maley, & Brumfield, 2014). Clines were modelled with either southern haplotype frequency (for COI) or mean admixture coefficients (*Q*‐values for microsatellites and SNPs) determined by STRUCTURE (Pritchard et al., 2000) against least‐cost distances among sampling sites. Clines were also modelled for each SNP independently based on allele frequency. For the SNP panel used in our present study, clines were modelled using *Q*‐value estimated from all available data given the limited differences between life stages and sampling year (see Supporting Information). Nonetheless, the genetic clines for 2015 juveniles, 2015 adults and 2011/2013 adults separately are presented in the Supplement along with the cline for the combined SNP data set (Supporting Information Figure S3). The results show that ML clines generally overlapped for these four data sets (except in parts of the southern range where sampling was limited in 2015). While the clines did shift southward between 2011/2013 and 2015, we found no significant differences observed in cline centres or widths among time periods and life stages (Supporting Information Table S3). However, when all data sets were combined (the full SNP data set with all life stages and years together), cline width was significantly larger than the cline width for 2011/2013 adult data set alone, and this may be attributed to the inclusion of finer‐scale sampling in the hybrid zone for later time periods. For all models, we calculated “coastal” distance from the southernmost site (Tuckerton, NJ; TKT) to each site using least‐cost distance in the R package *marmap* (Pante & Simon‐Bouhet, 2013). Least‐cost distances were estimated with depth restricted between 0 and 30 m to give a best estimate of distance along the shoreline. However, to allow movement across greater depths, maximum depth was increased to 60 m for the site located on Grand Manan Island, NB. The same was performed for sites located beyond Sydney Harbour, NS (SYH), because some of these sites were on islands (Brudenell River, PE, and Baie de Bassin, QC), and thus, we increased maximum depth for all sites in this region to provide distances consistent with *C. maenas* movement. At last, for sites in western NL, maximum depth was increased to 300 m to allow distance calculations to these sites (i.e., across the Laurentian Channel). The four sites in southeastern NL within Placentia Bay (NOH, BTH and FRH) and Fortune Bay (FTB) were excluded from cline analyses, as these sites could skew our analyses because they do not represent an area of natural hybridization between lineages (Blakeslee et al., 2010) and are beyond the linear coastline of the two invasion fronts.

In *hzar*, clines were fit with a null model and 15 different models that varied in the assumptions of the cline parameters (frequency intervals and exponential tails), where models assumed different combinations of (a) scaling of frequency intervals (fixed at 0 and 1, free scaling based on model estimates or no scaling and fit to observed values) and (b) fitting of exponential decay of the tails at the ends of the cline (neither tail, both tails, left tail only, right tail only or mirrored tails). Model selection was performed using corrected Akaike Information Criterion (AICc) scores, and ML cline centre and width were extracted from the best model. Clines were modelled with 95% credible cline region, and ML estimates for cline centre and width included two log‐likelihood low and high support limits. For comparisons between marker types and years, significant differences were determined based on two log‐likelihood support limits for the cline centre and width. For diploid markers, models were weighted by the effective number of alleles following a similar approach as Macholán et al. (2008) (see Supporting Information for full details of calculations). This weighting resulted in populations being given lower weights when they deviated more from Hardy–Weinberg equilibrium as suggested by Derryberry et al. (2014) when using *hzar*. For allele frequency clines for each SNP, the number of effective alleles was used to weight models, whereas the mean number of effective alleles across all loci was used to weight models for *Q*‐values for microsatellite and SNP data sets.

In addition, to quantify the temporal progression of the cline, the rate of change for the cline centre was calculated from its movement between each consecutive sample year for 2000, 2002, 2007 and 2015. These rates of change were then compared with predicted rates from a previous study modelling this from COI data (Pringle et al., 2011).

## 3 RESULTS

### 3.1 Population structure

Pairwise *F*_ST_ values were calculated for all adult and juvenile SNP samples collected in 2015 to evaluate differences between life stages. For all comparisons, *p*‐values were adjusted to control for false discovery rate (FDR). In general, *F*_ST_ was lowest among adult and juvenile samples collected from the same location (Supporting Information Figure S2A). Pairwise *F*_ST_ values were also lower among nearby locations relative to more distant locations (Supporting Information Figure S2A). For site‐specific comparisons between adults and juveniles, only two sites (Fortune Bay, NL; FTB and Hampton, NS; HMP) showed a significant genetic divergence between life stages (both *p *<* *0.05; Table 2). The limited differences between adult and juvenile samples were further visualized using a neighbour‐joining tree; adult and juvenile samples from the same site generally grouped close to each other on the tree, including FTB adults and juveniles (Supporting Information Figure S2B).

Next, pairwise *F*_ST_ values were calculated between adult samples collected from the same sites in 2011 and 2015, and no significant genetic divergence was found between years at the three sites (all *p*‐values >0.108; Supporting Information Table S2). As we found limited genetic divergence within sampling locations between different life stages or different years, samples were combined for all further analyses. Analyses of the pooled samples showed a mean pairwise *F*_ST_ between sampling sites of 0.108, ranging from 0 to 0.434 (Supporting Information Table S4). Higher *F*_ST_ values were generally observed between sites located in northern and southern regions. For example, mean pairwise *F*_ST_ values for more southern sites (TKT to HMP) and more northern sites (YRM to CNB) were 0.04 and 0.05, respectively, whereas the mean pairwise *F*_ST_ value between southern versus northern sites was 0.19.

**Figure 1:**
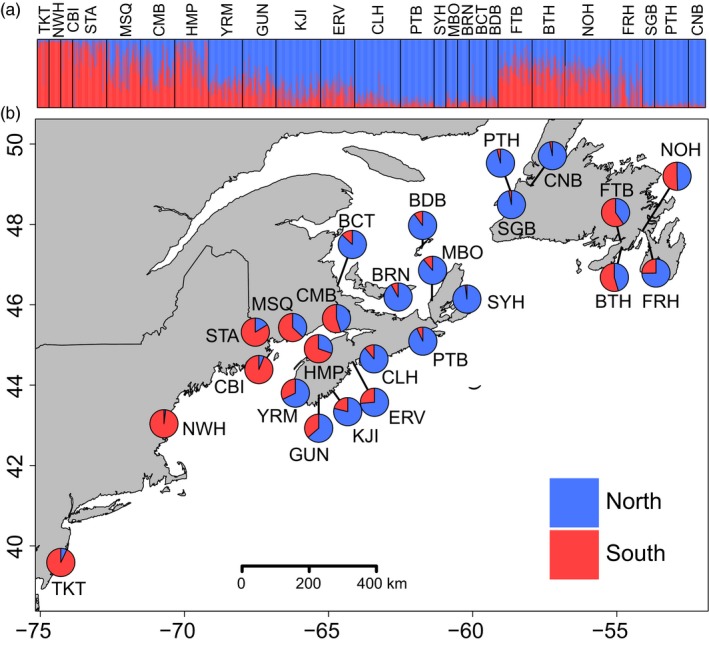
Results of Bayesian clustering analysis for 25 European green crab (*Carcinus maenas*) sampling locations using 96 collectively informative loci. (a) Individual assignment of crabs to two genetic clusters (north [blue]) and south [red]) where each individual is represented by a vertical bar indicating the proportion of membership to each cluster. (b) Map of sampling locations with the proportion of membership assigned to the two clusters for each site

Results from STRUCTURE revealed the distribution of two genetic clusters corresponding to the northern and southern lineages (Figure 1). Intermediate admixture coefficients were found from southern NB (at Musquash; MSQ) to southern NS (East River Point; ERV) consistent with the natural zone of secondary contact between the initial northern and southern invasions. Sites in southeastern NL also showed intermediate admixture coefficients. A neighbour‐joining tree of all samples showed two groups, where a division between northern and southern groups occurred along the coastline between Yarmouth, NS (YRM) and Hampton, NS (HMP) (Supporting Information Figure S4). The only exceptions were two southeastern NL sites (Boat Harbour, NL [BTH] and FTB), which both grouped with southern sites; however, bootstrap support was low for these nodes (see Supporting Information Figure S4).

### 3.2 Hybrid assignment

**Figure 2:**
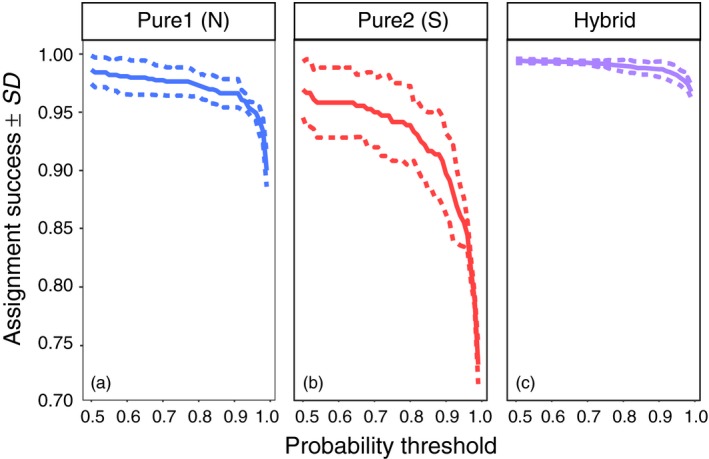
Mean (±*SE*) assignment success from nine simulated data sets (three simulated data sets with three replicates each) of European green crab (*Carcinus maenas*) for three genotype classes including (a) pure north, (b) pure south and (c) hybrid using the programs NEWHYBRIDS (Anderson, 2008) and *hybriddetective* (Wringe et al., 2017)

**Figure 3:**
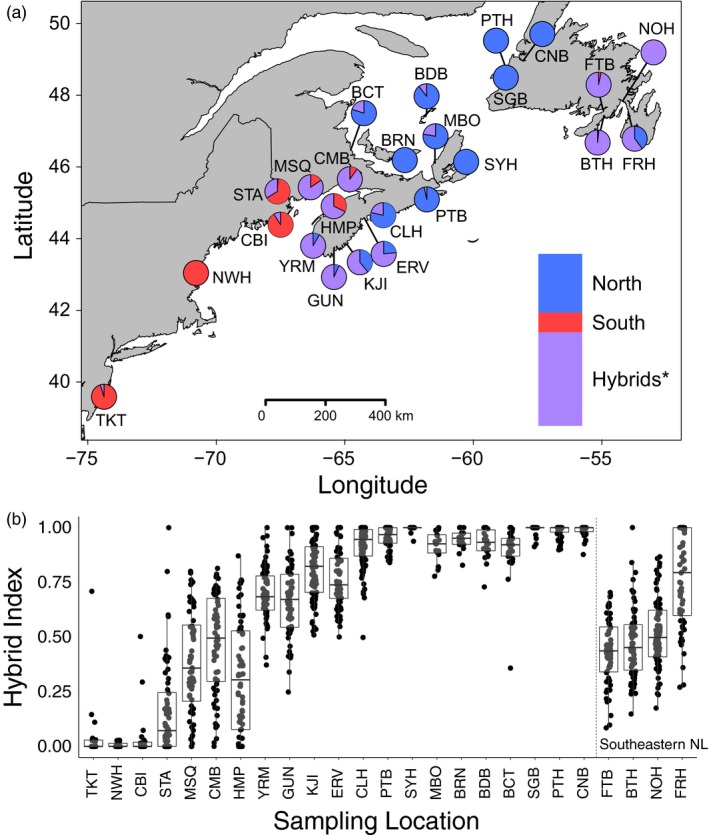
(a) Map of 25 European green crab (*Carcinus maenas*) sampling locations with the proportion of individuals assigned to pure or hybrid genotype classes by the program NEWHYBRIDS based on 96 informative SNPs. Map shows assignment to two pure (north and south) and a single hybrid class. Inset bar plots show the proportion of individuals assigned to each genotype class across all sites. The asterisk (*) denotes that all hybrids were assigned as recombinant hybrids (i.e., no first‐generation hybrids) using subsequent analyses in NEWHYBRIDS. (b) Boxplot of individual hybrid indices for sampling locations calculated using methods described by Buerkle (2005). Locations are arranged from south to north (Tuckerton, NJ to Corner Brook, NL) with known hybrid locations in southeastern NL shown separately. A hybrid index of 0 represents a pure south individual, whereas a hybrid index of 1 represents a pure north individual. Data points that fall within the upper and lower quartiles of the population are coloured grey, whereas those that fall outside these regions are coloured black

Our panel of 96 SNPs proved capable of accurately detecting both pure and hybrid individuals (see Supporting Information for details and Figure 2). Across the *C. maenas* range, individuals were first assigned to three possible genotype classes (pure north, pure south and hybrid), where approximately 93% of the genotyped individuals were assigned with a posterior probability greater than 0.85. Subsequent assignment of individuals into pure type, first‐generation hybrid (F_1_) or recombinant hybrid (i.e., later generation hybrids including F_2_ and backcrosses) classes revealed no F_1_ hybrids across the study range thus leaving all hybrids to be classified as recombinant hybrids (see Supporting Information for details; Supporting Information Figure S5B). The division between the pure northern and pure southern lineages occurred along the NS shoreline in the Bay of Fundy region between the sites HMP and YRM (Figure 3a). Following the coastline, the pure northern lineage appeared to be restricted to sites from YRM eastward, with the exception of a single northern individual found in St. Andrew's, NB (STA) (Figure 3a). The pure southern lineage was restricted to sites from HMP westward, with the exception of two southern individuals found in FTB (Figure 3a). The percentage of hybrids within each site ranged from 0% to 100%, with extensive hybridization observed at sites located from the Bay of Fundy region (in southern NB and NS) and extending eastward along the south shore of NS to East River Point (ERV) (Figure 3a). Extensive hybridization was also detected in southeastern NL (Figure 3a).

Individuals were also assigned maximum‐likelihood hybrid indices using GENODIVE (Meirmans & Van Tienderen, 2004) where results agreed with NEWHYBRIDS assignment. Hybrid indices showed variation across most site locations, with more hybrids observed between STA and ERV, as well as in southeastern NL (Figure 3b). Further, in southeastern NL, the limited number of pure individuals in most locations supports the introduction of admixed individuals to this region (Blakeslee et al., 2010; Jeffery et al., 2017a). The large variation in hybrid indices within many sites is consistent with the assignment of recombinant hybrids rather than first‐generation hybrids in the hybrid regions.

*INTROGRESS* (Gompert & Buerkle, 2010) provided further support that hybrids represented recombinant hybrids; however, only a few individuals appear to show signatures reflective of potential third‐generation hybrids (see Supporting Information Figure S6). The hybrid zone in NB/NS showed similar levels of introgression as the introduced hybrid region in southeastern NL (Supporting Information Figure S6).

### 3.3 Temporal changes to the genetic clines

**Figure 4:**
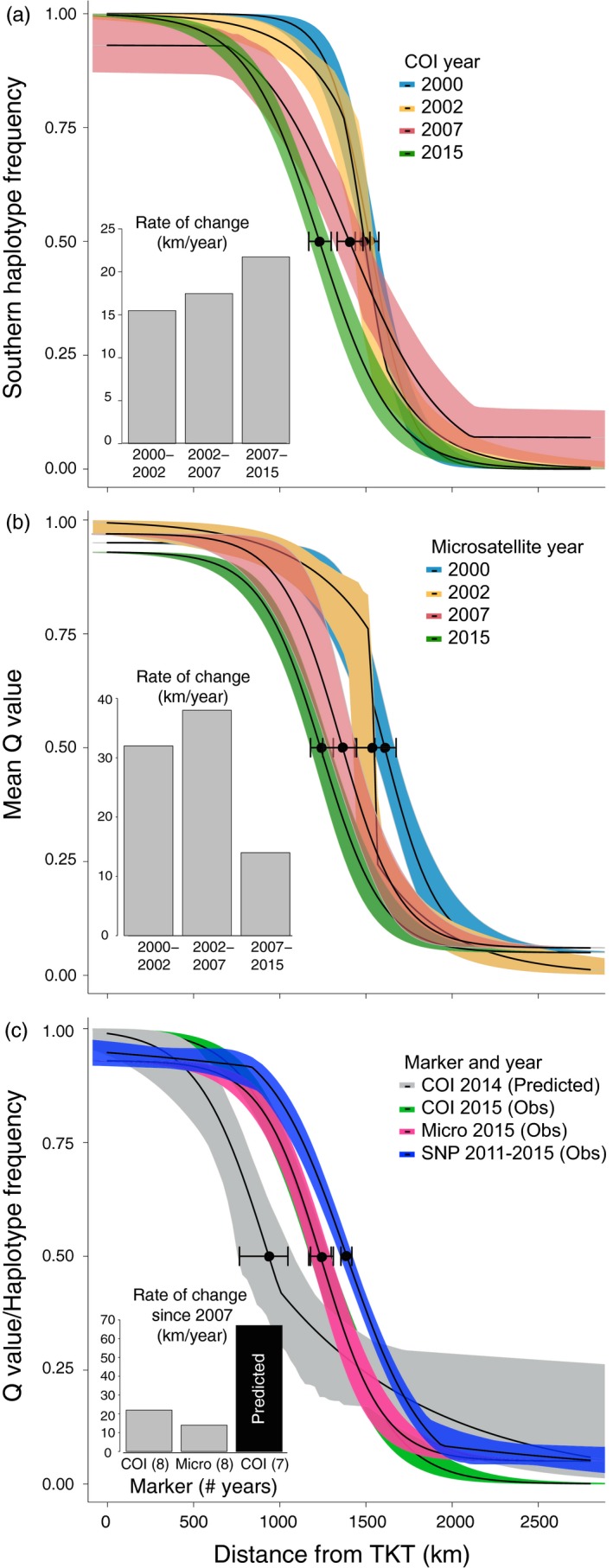
Maximum‐likelihood (ML) genetic clines for European green crab (*Carcinus maenas*) for different time points and genetic markers where clines were modelled using *hzar* (Derryberry et al., 2014) with either the southern haplotype frequency (mitochondrial marker COI) or mean admixture coefficient (*Q*‐value; microsatellite and SNP markers) against the distance from the southernmost site in Tuckerton, New Jersey (TKT). Clines are shown with their associated fuzzy cline region (95% credible cline region) and the ML estimate of cline centre is indicated (dot) with two log‐likelihood low and high estimates (whiskers). Panel insets show the rate of change (distance per year) for the estimated ML cline centre for each time period. Temporal genetic clines are shown for (a) COI and (b) microsatellite markers. In panel (c), the predicted 2014 genetic cline from Pringle et al. (2011) is shown relative to the recent genetic clines for all markers including the panel of 96 informative SNPs. Note that sites in southeastern Newfoundland were excluded from the analyses. Clines for microsatellite and SNP data were weighted based on their effective number of alleles as described in the Section 2

**Figure 5:**
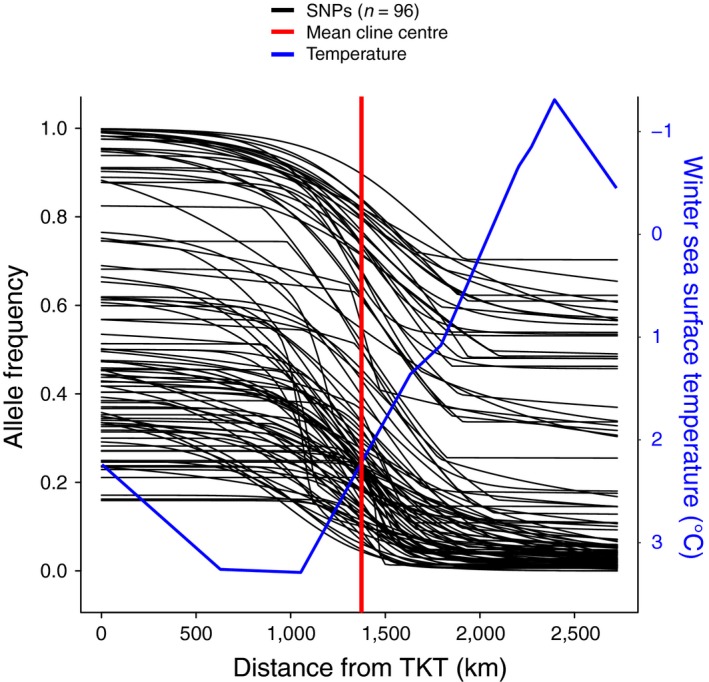
Maximum‐likelihood (ML) genetic clines for European green crab (*Carcinus maenas*) for 96 single nucleotide polymorphisms (SNPs) where clines (black lines) were modelled using *hzar* (Derryberry et al., 2014) with allele frequency against the distance from Tuckerton, New Jersey (TKT) for each site. The mean ML estimate of cline centre is indicated by the red line. Mean winter sea surface temperature (blue line) from 2018Jeffery et al. (2018) was added on an inverse scale on the right‐hand axis for comparison against cline models. Clines for each SNP were weighted based on their effective number of alleles as described in the Section 2

Clines were modelled for several time periods between 2000 and 2015 for COI and microsatellite markers (Figure 4a,b) across all sites with the exception of locations in southeastern NL. Results of all maximum‐likelihood (ML) cline models are provided in Table 3 and Figure 4. Both COI and microsatellite markers showed a southward progression of the clines over time, where cline centres in 2000 and 2015 differed significantly within each marker type (Figure 4a,b; Table 3). In addition, the cline width increased significantly between 2000 and 2015 for the COI marker (Table 3). The width of the cline was not significantly different between 2000 and 2015 for microsatellites; however, the width of the cline increased significantly between 2002 and 2015 (Table 3). Clines for SNPs were modelled based on *Q*‐value as well as allele frequency for each SNP independently (Figures 4 and 5). The mean cline for SNP allele frequencies (averaged across ML clines for all SNPs) did not differ significantly in cline centre or width from the cline modelled for SNP *Q*‐value as well as COI and microsatellite markers in 2015. Given that *C. maenas* genetic clines have recently been associated with spatial variation in cold temperatures, average winter sea surface temperature data for 11 sites from Jeffery et al. (2018) was included in Figure 5 for comparison against the SNP allele frequency clines.

**Table 3:** Results and cline parameters for best fitting model from *hzar* analyses for each marker type and year

Marker and year	AICc	Centre estimates (km)		Width estimates (km)		Best model (scaling, tails)
		ML	2LL low–high	ML	2LL low–high	
Microsatellites						
2000	24.42	1609^a^	1,531–1,675	619^abc^	411–947	Fixed scaling, neither tail
2002	22.78	1544^abc^	1,436–1,551	43^d^	21–401	No scaling, mirror tails
2007	6.53	1356^cde^	1,252–1,448	694^abcd^	393–955	Fixed scaling, neither tail
2015	7.92	1244^e^	1,178–1,310	708^bc^	562–865	Fixed scaling, neither tail
COI						
2000	18.61	1522^ab^	1,467–1,574	481^cd^	370–638	Fixed scaling, neither tail
2002	56.38	1491^bc^	1,438–1,523	395^cd^	91–606	Fixed scaling, mirror tails
2007	91.43	1404^bcd^	1,333–1,482	1100^a^	868–1,387	Fixed scaling, mirror tails
2015	32.04	1230^e^	1,168–1,298	849^ab^	705–1,037	Fixed scaling, neither tail
Prediction (2014)	10.38	936^f^	766–1,047	828^abcd^	400–1,294	No scaling, right tail only
SNP						
*Q*‐value	53.77	1389^d^	1,355–1,418	925^ab^	830–1,052	No scaling, mirror tails
Allele frequency*	—	1374^cde^	1,272–1,463	1006^ab^	701–1,442	—

*Notes*.

Maximum‐likelihood (ML) cline centre and width are provided with their two log‐likelihood (2LL) low and high support limits. Significant differences (based on 2LL high and low) are denoted by different letters, where overlapping cline centre and width estimates represent clines that are coincident and concordant, respectively. For single nucleotide polymorphisms (SNPs), mean cline parameters are provided based on clines for all 96 SNPs. Models for SNPs and microsatellites were weighted by effective number of alleles (see Section 2).

*Mean of all SNP allele frequency models.

The results of the most recent genetic clines modelled for all marker types, including the SNP data (*Q*‐value and allele frequency for each SNP), show that the clines have not progressed as far south as previously predicted by Pringle et al. (2011) (Figure 4c). In specific manner, for all marker types, fewer northern genotypes were observed near the cline centre predicted by neutral advective models in Pringle et al. (2011), suggesting northern individuals have not shifted southward to the extent predicted (Table 3; Figure 4c). The estimates of cline centre for all marker types differed significantly from the predicted cline centre; however, the cline width did not differ significantly between observed and predicted clines. Therefore, observed and predicted clines are noncoincident but concordant.

Microsatellite and COI markers showed similar clines (i.e., coincident and concordant) for 2015 (see Supporting Information Figure S7 for a map of current population structure for both markers), but the SNP *Q*‐value data showed less of a southward shift over the range relative to these markers (Figure 4c). However, when data for the SNP panel were separated by year and life stage (see Supporting Information Table S3 and Supporting Information Figure S3), a temporal shift in cline centre and an increase in cline width were observed over time (i.e., from adults in 2011–2013 to adults in 2015 to juveniles in 2015); however, confidence intervals overlapped indicating no significant difference between estimates (Supporting Information Table S3) although differences in sampling range could influence this interpretation because 2015 adults and juveniles did not contain samples located south of St. Andrews, NB (STA).

Furthermore, although the SNP *Q*‐value cline showed less of a shift than COI and microsatellites, when we examined individual SNP loci (allele frequencies), many SNP clines were coincident with the 2015 COI and microsatellite cline (45% and 51%, respectively) (Supporting Information Figure S8A). However, 48% and 43% of the SNP clines were noncoincident and located further northward relative to the COI and microsatellite clines, respectively (Supporting Information Figure S8A). For cline widths, the widths of 64% and 65% of the SNP clines were concordant with COI and microsatellite clines (Supporting Information Figure S8B). Variation in ML cline centre and width for SNPs were not significantly related to *F*_ST_ values of the SNPs (centre *p *=* *0.09; width *p *=* *0.27) (Supporting Information Figure S8).

We also quantified the rate of change (distance per year) for the centre of the clines, and different marker types revealed different patterns. For microsatellites, the rate of change decreased between 2000 and 2015 (Figure 4b), whereas the rate of change increased for the COI marker over the 15‐year period (Figure 4a). Nonetheless, microsatellite and COI markers in 2015 showed similar clines to each other (i.e., no difference in cline width or centre), and the rate of change observed for both clines since 2007 were slower than the rate that was predicted by Pringle et al. (2011) during a similar time period. We estimated the rate of change for the cline centre to be approximately 14 and 21 km/year for the observed microsatellite and COI data, respectively, whereas the rate of change predicted by the neutral Pringle et al. (2011) model for this time (observed 2007 COI to predicted 2014 COI) was more than triple those rates (Figure 4c).

## 4 DISCUSSION

The repeated invasion of *C. maenas* into eastern North America provides an unprecedented opportunity to explore secondary contact as it occurs and examine the factors that promote temporal stability of genetic clines. Consistent with previous studies, clinal analyses revealed two genetically distinct groups of *C. maenas* corresponding to southern and northern lineages derived from two independent introductions (Blakeslee et al., 2010; Darling et al., 2014; Jeffery et al., 2017a; Pringle et al., 2011; Roman, 2006). Our results confirm the presence of extensive hybridization in the zone of secondary contact in southern Nova Scotia and the Bay of Fundy (Jeffery et al., 2017a) with potentially diminishing direct interactions of the pure lineages (i.e., no first‐generation hybrids were detected). In an interesting manner, displacement of the clines was slower relative to expectations of passive dispersal (Pringle et al., 2011), which may support the hypothesis that other mechanisms, such as selection, nonrandom dispersal and/or other demographic processes, are influencing cline dynamics. Our results demonstrate the difficulties in forecasting invasion dynamics and highlight the possible interaction of multiple processes including dispersal, hybridization and selection in regulating invasion success.

### 4.1 Temporal progression of the clines

Our data set provides an opportunity to explore the evolution of the *C. maenas* clines in eastern North America, building on previous studies examining temporal cline dynamics between 2000 and 2007 (Darling et al., 2014; Pringle et al., 2011). We observed a southward progression of the cline centre over time in both microsatellite and mitochondrial (COI) markers. Temporal changes in clines have been observed across many systems, and a wide range of mechanisms can drive such shifts including climate change, habitat alterations, hybridization, skewed sex ratios, mate choice, and competitive exclusion (reviewed in Buggs, 2007). In *C. maenas*, ongoing hybridization and advective dispersal is likely resulting in continued cline shifts as predicted by a neutral model of advective dispersal (Pringle et al., 2011); however, it is noteworthy that clines for all marker types displayed deviations from those model predictions. Consistent with Darling et al. (2014), this may suggest that mechanisms other than larval dispersal alone may be contributing to temporal changes in the clines (discussed below). Instead, it may suggest the involvement of neutral processes not accounted for by Pringle et al. (2011), such as atypical hydrodynamic features that drive nonrandom larval dispersal and can thus generate steep clines in the absence of selection (Hare, Guenther, & Fagan, 2005).

In an interesting manner, we also observed differences in the speed of cline movement between the COI and microsatellite markers. Both marker types displayed a slower southwards progression than predicted by the neutral, advective dispersal model (Pringle et al., 2011), yet the progression of the microsatellite cline was less than the COI cline over time (as also observed in the 2000–2007 data in Darling et al., 2014). Discordance between nuclear and mitochondrial markers is not uncommon, although the mechanisms responsible are often difficult to ascertain and sex‐biased processes, mito‐nuclear incompatibility and/or adaptive introgression are frequently proposed drivers of mito‐nuclear discordance (Toews & Brelsford, 2012; Wolff, Ladoukakis, Enríquez, & Dowling, 2014). Specific to *C. maenas*, Darling et al. (2014) suggested that gene surfing (Currat, Ruedi, Petit, & Excoffier, 2008) and genetic “stickiness” (i.e., protection from Allee effects through hybridization) (Mesgaran et al., 2016) likely contributed to the rapid bi‐directional expansion of the mitochondrial cline relative to nuclear markers, and these potential mechanisms may also explain temporal changes observed here. Further, the enhanced influence of drift due to haploidy, maternal inheritance and background selection in the absence of recombination could also result in different rates of movement for the COI marker relative to diploid markers. Despite differences between marker types, the COI marker represents the most diagnostic marker for tracking the distribution of the genetic lineages, thus providing more weight to our conclusions regarding this species’ spread over time (Darling et al., 2014; Pringle et al., 2011; Roman, 2006). Our study thus provides a rare, long‐term perspective of species invasion distribution and spread over time, from its inception to the present.

### 4.2 Potential mechanisms contributing to current cline dynamics

All marker types resolved a common spatial genetic pattern, where current clines for COI, microsatellites, and SNP allele frequencies (mean overall loci and many individual SNPs) overlapped in cline centre and width, and the widths of these clines were also concordant with that of the SNP cline based on *Q*‐value. However, current clines generated for all marker types did not coincide with previous predictions potentially resulting from asymmetric connectivity and/or limitations of previous forecasting models (Pringle et al., 2011). Instead, deviations from predictions may implicate selective processes in shaping the current clines where cline dynamics may be influenced by physiological differences related to environmental conditions such as temperature (Jeffery et al., 2018; Tepolt & Somero, 2014). Latitudinal clinal patterns exist in the native range of *C. maenas* (Roman & Palumbi, 2004), and differences in thermal tolerance (i.e., cardiac threshold) exist between crabs from northern and southern parts of the native range, with higher latitude populations being less tolerant of warmer conditions and *vice versa* (Tepolt & Somero, 2014). While similar thermal differences exist between parts of the invaded range, it is unclear whether these physiological differences reflect adaptive divergence or genomewide divergence carried over from the native range that has been maintained through neutral processes (Tepolt & Somero, 2014). Recent work suggests associations between genetic structuring and winter sea surface temperature (Jeffery et al., 2018) that are the result of recent secondary contact that has resulted in a hybrid zone that coincides with a temperature gradient in the Atlantic Ocean (Bierne, Welch, Loire, Bonhomme, & David, 2011) (see Figure 5). In this case, the coupling of endogenous (genetic incompatibilities) and exogenous (environmental) barriers may drive signals of local adaptation when genetic incompatibilities may be more likely (Bierne et al., 2011). Here, we cannot determine to what extent this coupling hypothesis (*sensu* Bierne et al., 2011) influences the *C. maenas* clines, although genetic incompatibilities between lineages appear minimal. In fact, the temporal broadening of the cline widths across marker types suggests that selection against hybrids is weak (Sotka & Palumbi, 2006) and this is evident by large numbers of later generation hybrids in our data set. Further, the presence of an apparently stable admixture zone in southeastern NL suggests the absence of strong selection effects against hybrids as well as limited selection related to thermal tolerance in these colder waters.

Indeed, patterns of hybridization in the zone of secondary contact may provide insight into the mechanisms that may be operating to slow the progression of the clines relative to predictions (Pringle et al., 2011). Samples from sites located between St. Andrews, NB (STA) to East River, NS (ERV) showed extensive hybridization between lineages, consistent with secondary contact. However, only later generation recombinant hybrids were observed, where no F_1_ hybrids were found across the entire range, even though our panel of 96 SNPs proved capable of distinguishing F_1_s in simulated data sets. Our results are consistent with those of Jeffery et al. (2017a), where primarily later generation hybrids were observed and only a single F_1_ hybrid individual was detected across the range in their study. The absence of F_1_ hybrids may suggest diminishing direct contemporary contact between the two invasion fronts, potentially resulting from weak selection against hybrids (i.e., as evidenced by the persistence of an admixture region in southeastern NL) and an expanding hybrid zone that impedes the interaction of pure types (Jeffery et al., 2017a).

### 4.3 Future of the green crab clines

Our study indicates that the genetic clines of *C. maenas* have progressed southward slower than predicted by Pringle et al. (2011), perhaps implicating processes such as environmentally associated selection, later generation hybrid advantage, nonrandom larval dispersal and demographic processes in shaping cline dynamics in this system. Experimental studies are needed to resolve the role of these mechanisms in influencing *C. maenas* clines. Moreover, future cline dynamics may be further altered by human‐mediated global change (Taylor, Larson, & Harrison, 2015). For example, climate change associated ocean warming and acidification could lead to range shifts and expansions in *C. maenas* lineages (Compton, Leathwick, & Inglis, 2010; Gibson, Atkinson, Gordon, Smith, & Hughes, 2011). Further, recent predictions from Stanley et al. (2018) suggest that the genetic cline centres of multiple species in the Northwest Atlantic, including *C. maenas*, will shift northward under future climate scenarios with both lineages of *C. maenas* experiencing increases in the extent of suitable habitat. In addition, the anthropogenic transport of individuals via ship traffic and other anthropogenic vectors (e.g., see Blakeslee et al., 2010; Cohen, Carlton, & Fountain, 1995; Fowler et al., 2016) could also influence the future distribution and population structure of *C. maenas*. It also remains unclear how *C. maenas* distributions could be influenced by competition with the more recent invader the Asian shore crab (*Hemigrapsus sanguineus*) (Lord & Williams, 2017). Altogether, several factors may contribute to ongoing changes in spatial genetic structure, and given the reported differences in behaviour, physiology and reproduction between *C. maenas* from different regions (Best et al., 2017; Rossong et al., 2011; Tepolt & Somero, 2014), temporal genetic sampling over 5‐ to 10‐year intervals should be implemented to monitor these potential changes and help facilitate more appropriate management strategies.

## 5 CONCLUSIONS

In our study, multiple genetic marker types suggest a slowed southward movement of *C. maenas* genetic clines over time counter to dispersal‐based predictions. It is likely that both selective and neutral processes shape the current clines, and future work using complex realistic models of dispersal and experimental work on quantifying the competitive ability and physiology of hybrid and genetically pure individuals are necessary to better understand the processes restricting the range limits of each lineage and help predict future dynamics of the cline. In addition, climate change and human‐mediated transport may lead to continued alterations and range shifts in *C. maenas* distributions. Our study highlights the current challenges and complexities in forecasting invasion dynamics and emphasizes the importance of temporal monitoring using genetic markers to understand the spread of invasive species and inform management decisions.

## CONFLICT OF INTEREST

None declared.

## DATA ARCHIVING

Data available from the Dryad Digital Repository: https://doi.org/10.5061/dryad.130vb65.
