# Supplementary material for: Temporal dynamics of genetic clines of invasive European green crab (Carcinus maenas) in eastern North America
Source: Evol Appl. 2018 Jun 28;11(9):1656–70. doi: 10.1111/eva.12657 (PMC6183463; doi:10.1111/eva.12657)
Supplement: Supplementary file 1 [file EVA-11-1656-s001.docx]

# Supplementary

## Supplemental methods

### Hybrid assignment

To assign hybrids within our sample, we used the program NEWHYBRIDS v1.1 (Anderson, 2008), where we first tested the ability of our 96 SNPs to accurately identify hybrids. Guided by results from STRUCTURE, we identified pure south and pure north individuals from our sample based on admixture coefficients (Q values > 0.90 or < 0.10, respectively). Using *hybriddetective* (Wringe et al., 2017) genotypes of pure north and south lineage individuals were used to create nine datasets (three simulated datasets with three replicates each) that simulated genotypes of individuals belonging to six genotype classes including two pure parental classes (south and north) and four hybrid classes (first generation hybrid (F_1_), second generation hybrid (F_2_), and both backcrosses). Simulated datasets were run in NEWHYBRIDS in parallel through the R package *parallelnewhybrid* (Wringe et al., 2017) using 100,000 burn-in and 300,000 MCMC iterations. To determine the accuracy of assignments, *hybriddetective* was used to quantify correct assignment within the simulated datasets.

After accuracy of assignment was verified, all crab samples were run in NEWHYBRIDS along with simulated pure north and south individuals with their known classification (‘z’ option) included in the analysis (Anderson, 2003). Only individuals that were classified to a genotype class with a posterior probability >0.85 were used in our analysis. Posterior probabilities were primarily evaluated for three genotype classes (pure north, pure south, and hybrid); however we subsequently evaluated six genotype classes (pure north, pure south, F_1_, F_2_, and both backcrosses). We chose to only report these hybrids as two groups: first generation (F_1_) and recombinant hybrids (F_2_ and backcrosses). We have classified second-generation hybrids as recombinant hybrids because we recognize that additional later generation hybrid classes (e.g. F_3_ and later generations) may be present in the populations however assignments can be unreliable for these hybrids and were thus not assayed. Results from NEWHYBRIDS were validated by calculating hybrid indices (ranging from 0 (pure south) to 1 (pure north)) for individuals in each population using maximum-likelihood methods described by Buerkle (2005) implemented in GENODIVE (Meirmans & Van Tienderen, 2004), where the same simulated pure north and pure south individuals (as above) were used as the two reference populations.

### Mitochondrial DNA (COI) sampling and sequencing

Mitochondrial genotypes for European green crab (*Carcinus maenas*) young-of-the-year (YOY) collected in 2000, 2002, and 2007 were compiled from previous studies (Roman, 2006; Pringle et al., 2011) (see Table S5 for sample and location information). During summer 2015, following the same protocol as previous years, *C. maenas* YOY were hand-collected in the intertidal zone by turning over rocks and cobble (see Table S5 for locations). In some New England and mid-Atlantic locations, *C. maenas* YOY are much less abundant, or are absent, in the intertidal zone due to the presence of another invasive crab, *Hemigrapsus sanguineus*. In these locations, we were limited in the number of crabs we could hand-collect and therefore employed collapsible crab traps to obtain crabs. These crabs were typically adults, and any locations where only adults or both adults and juveniles were collected are indicated in Table S5. Even so, these locations are still below the major center of the cline (see Figure 5) and thus would be mostly representative of the earlier 1800s introduction; i.e., the southern genotype (Roman, 2006; Darling et al., 2014). In the lab, crabs were dissected for their gill tissue, which was preserved (frozen or in 95% ethanol) until DNA extraction using a standard CTAB protocol (France et al., 1996). A 502-bp region of the mitochondrial cytochrome oxidase I (COI) gene fragment was amplified with primers and protocols designed for *Carcinus* (Roman & Palumbi, 2004; Blakeslee et al., 2010; Pringle et al., 2011). Purified amplicons were sequenced at MacrogenUSA (Rockville, Maryland, USA). COI sequences were inspected for ambiguities using Geneious8.1.7 (Biomatters Ltd., Auckland, New Zealand) and aligned without gaps using ClustalW (Larkin et al., 2007).

### Microsatellite genotyping

For microsatellites, *C. maenas* YOY were collected in the same way as described above for COI sampling (see Table S5 for locations). Microsatellite genotypes at nine microsatellite markers for the years 2000, 2002 and 2007 were compiled from previous studies (Blakeslee et al., 2010; Darling et al., 2014). *C. maenas* YOY collected in 2015 were genotyped at 11 previously described microsatellite loci including Cma01EPA, Cma02EPA, Cma03EPA, Cma04EPA, Cma05EPA, Cma08EPA, Cma09EPA, Cma14EPA (Tepolt et al., 2006), Cma16EPA (Darling et al., 2008), SP107 and SP495 (Pascoal et al., 2009). Multiplex polymerase chain reactions (PCRs) were performed where the full protocol for multiplex reactions are described in Jeffery et al. (2017a). Each reaction was performed in a volume of 10 µl which consisted of 10 ng of template DNA, 1X Type-it Multiplex PCR Master Mix (Qiagen, Toronto, Canada), and 0.1–1.0 µM of each primer. The loci were amplified with the following protocol: initial denaturation step for 5 minutes at 95°C, followed by 28 cycles of 30 seconds at 95°C, 5 minutes at reaction-specific annealing temperature (see Jeffery et al. (2017a) for temperatures), 30 seconds extension at 72°C, then a final extension step of 30 minutes at 60°C. Following amplification, size fractionation of PCR products was completed using an AB3130xl capillary electrophoresis system (Life Technologies) with a 36 cm array and POP7 polymer, and GeneScan 500 LIZ (Life Technologies) was included as an internal size standard with each sample. GeneMapper version 5.0 (Life Technologies) was then used to determine the size of all fragments (alleles) from resulting electropherograms. Intra- and inter-plate controls were included on each 96-well DNA plate and these controls were used to identify errors made during sample processing.

### COI and microsatellite data analyses

All COI sequences were collapsed into haplotypes using TCS1.21 (Clement, Posada, & Crandall, 2000) and the frequency of southern haplotypes within each population was determined (see Table S5) and used for subsequent clinal analyses. For the four sites where adults and juveniles were collected together, adult and juvenile haplotypes were combined to determine the haplotype frequency for the total population. For microsatellite data, the optimal number of genetic clusters (K) was determined using Bayesian inference in the software STRUCTURE ver. 2.3.4 (Pritchard, Stephens, & Donnelly, 2000) with Evanno’s ∆*K* method (Evanno, Regnaut, & Goudet, 2005). The admixture coefficient (Q-value) of each individual was calculated with the same software, and a mean Q-value calculated for each site (see Table S5).

### Temporal changes in the genetic clines

#### Weighting of *hzar* cline models

In *hzar*, clines for diploid markers were weighted by the effective number of alleles following a similar approach as Macholán et al. (2008). For each locus within each year, we calculated effective number of alleles using the equation:

Effective number of alleles = 2N / (2N*F*_ST_)+(1-*F*_ST_)(1+|*F*_IS_|)

where N is the number of individuals**,** *F*_ST_ represents a measure of genetic relatedness within the population across all loci and *F*_IS_ is a measure of excess/deficit of heterozygotes calculated for each locus within the population (Macholán et al., 2008). The equation from Macholán et al. (2008) was modified because we assumed *F*_ST_ within a population was equivalent to 0 as we expect individuals to be genetic similar within a site. Therefore the equation was reduced to:

Effective number of alleles = 2N / (1+|*F*_IS_|)

This equation results in populations being given lower weight in the model when they deviate more from Hardy-Weinberg equilibrium as suggested when using *hzar* (Derryberry et al., 2014). The R package *diveRsity* (Keenan et al., 2013) was used to calculate *F*_IS_ for each locus within each population and year. In the case that *F*_IS_ could not be calculated because all individuals were homozygous for the same allele, *F*_IS_ was given a value of 1. Effective number of alleles for each SNP were used to weight each SNP model for allele frequency clines and the mean effective number of alleles across all loci were used for weighting Q-value clines for microsatellite and SNP datasets.

## Supplemental results

### Accuracy of hybrid assignment

Accuracy of hybrid assignment for our panel of 96 SNPs was tested using nine simulated multigenerational hybrid datasets. On average, when evaluating the simulated datasets for the presence of three genotype classes (pure south, pure north, and hybrid), more than 90% of simulated individuals in each class could be assigned successfully at a posterior probability threshold of 0.85 (Figure 2). When six genotype classes in the simulated datasets were evaluated, assignment success of specific hybrid classes were lower and the range was approximately 60% for F_2_ hybrids, 70% for F_1_ hybrids and southern backcrosses, and 85% for northern backcrosses (Figure S5A). Given that assignment to additional generation hybrids (e.g., F_3_) was not explored in our study and it is possible that hybrids could represent such later generations, thus we considered all second generation (F_2_ and backcrosses) as a single group of “recombinant hybrids”.

#### Recombinant hybrid assignment

For our primary hybrid assignment we examined three genotype classes (pure south, pure north and hybrid). However, we subsequently divided hybrids into either first generation (F_1_) or recombinant hybrid (i.e., F_2_ and backcrosses) genotype classes, where 80% of our sampled individuals could be assigned to a genotype class using a posterior probability threshold of 0.85. No first generation (F_1_) hybrids were detected leaving only later generation hybrids (i.e. second generation hybrids (F_2_) and backcrosses) observed in our study (Fig. S5B).

## Supplemental tables

**Table S1.** Panel of 96 high-*F*_ST_ single nucleotide polymorphisms (SNPs) with low linkage disequilibrium selected based on green crabs (*Carcinus maenas*) across 11 populations previously genotyped by Jeffery et al. (2017) (see Figure S1 for distribution of all SNPs from their study). The table demonstrates the diagnostic nature of the panel where locus-specific *F*_ST_ values were calculated between the northernmost (Corner Brook, Newfoundland) and southernmost (Tuckerton, New Jersey) sites in our study.

| **Pairwise *F*_ST_ between north and south for informative SNPs** | | | | | | | |
| --- | --- | --- | --- | --- | --- | --- | --- |
| **SNP** | ***F*_ST_** |  | **SNP** | ***F*_ST_** |  | **SNP** | ***F*_ST_** |
| 10292_44 | 0.408 |  | 16189_33 | 0.156 |  | 22350_33 | 0.192 |
| 10920_32 | 0.581 |  | 16218_40 | 0.301 |  | 22610_31 | 0.505 |
| 11149_45 | 0.421 |  | 16247_39 | 0.203 |  | 22959_37 | 0.422 |
| 11183_46 | 0.363 |  | 16385_53 | 0.202 |  | 23025_52 | 0.309 |
| 11296_47 | 0.330 |  | 16668_29 | 0.304 |  | 2311_48 | 0.398 |
| 11791_50 | 0.561 |  | 16732_33 | 0.365 |  | 2384_34 | 0.380 |
| 11874_55 | 0.298 |  | 16814_40 | 0.071 |  | 2448_38 | 0.072 |
| 11919_29 | 0.380 |  | 17236_59 | 0.540 |  | 247_44 | 0.409 |
| 11977_29 | 0.191 |  | 17585_56 | 0.412 |  | 2560_51 | 0.498 |
| 12304_35 | 0.203 |  | 17670_42 | 0.497 |  | 316_30 | 0.446 |
| 12476_41 | 0.802 |  | 17710_45 | 0.202 |  | 3372_54 | 0.340 |
| 126_50 | 0.341 |  | 17976_26 | 0.324 |  | 3426_47 | 0.255 |
| 12932_29 | 0.334 |  | 18013_31 | 0.458 |  | 3512_30 | 0.302 |
| 13017_32 | 0.246 |  | 18065_42 | 0.250 |  | 375_50 | 0.446 |
| 13254_43 | 0.328 |  | 18733_28 | 0.233 |  | 3866_57 | 0.473 |
| 13707_31 | 0.406 |  | 18748_51 | 0.268 |  | 3995_28 | 0.677 |
| 13897_52 | 0.457 |  | 18826_38 | 0.283 |  | 4800_32 | 0.274 |
| 13957_36 | 0.352 |  | 19004_55 | 0.745 |  | 519_59 | 0.288 |
| 1408_38 | 0.493 |  | 19223_31 | 0.373 |  | 5317_50 | 0.412 |
| 14302_27 | 0.223 |  | 19364_32 | 0.473 |  | 5477_52 | 0.269 |
| 14401_42 | 0.335 |  | 19415_46 | 0.147 |  | 611_50 | 0.180 |
| 14448_44 | 0.396 |  | 19416_34 | 0.259 |  | 6233_54 | 0.211 |
| 14577_55 | 0.239 |  | 19473_51 | 0.255 |  | 6666_45 | 0.385 |
| 15053_43 | 0.443 |  | 19528_33 | 0.174 |  | 6870_54 | 0.354 |
| 15192_29 | 0.237 |  | 20060_32 | 0.475 |  | 6974_55 | 0.251 |
| 1567_49 | 0.334 |  | 20194_56 | 0.298 |  | 7021_47 | 0.479 |
| 15709_29 | 0.175 |  | 20497_38 | 0.112 |  | 705_31 | 0.156 |
| 15749_49 | 0.177 |  | 20773_31 | 0.515 |  | 7399_35 | 0.335 |
| 15757_47 | 0.206 |  | 21003_54 | 0.173 |  | 7959_47 | 0.318 |
| 15911_57 | 0.411 |  | 21142_52 | 0.372 |  | 8212_44 | 0.396 |
| 16033_32 | 0.430 |  | 21186_53 | 0.291 |  | 8262_39 | 0.480 |
| 16081_33 | 0.545 |  | 21615_38 | 0.287 |  | 9325_49 | 0.407 |
| **Mean *F*_ST_ = 0.344** | | | | | | | |

**Table S2.** Pairwise *F*_ST_ with corresponding significance (*P* values) for European green crab (*Carcinus maenas*) adults collected at the same sampling location during different sampling years (2011 and 2015).

| **Location** | **2011-2015 *F*_ST_** | ***P* value** |
| --- | --- | --- |
| KJI | 0.0030 | 0.1587 |
| CLH | 0.0039 | 0.1078 |
| NOH | 0.0034 | 0.1249 |

|  | **Centre estimates (km)** | |  | **Width estimates (km)** | |
| --- | --- | --- | --- | --- | --- |
| **Life stage and year** | **ML** | **2LL low-high** |  | **ML** | **2LL low-high** |
| 2011-2013 Adults | 1599^ab^ | 1378-1632 |  | 123^a^ | 10-613 |
| 2015 Adults | 1480^a^ | 1423-1527 |  | 172^ab^ | 162-920 |
| 2015 Juveniles | 1444^ab^ | 1391-1494 |  | 717^ab^ | 570-915 |
| All SNP data | 1389^b^ | 1355-1418 |  | 925^b^ | 830-1052 |

**Table S3.** Results and cline parameters for best fitting model from *hzar* analyses for single nucleotide polymorphism (SNP) datasets separated by life stage and sampling year. Maximum likelihood (ML) cline center and width are provided with their two log-likelihood (2LL) low and high support limits. Significant differences (based on 2LL high and low) are denoted by different letters, where overlapping cline centre and width estimates represent clines that are coincident and concordant, respectively.

|  | TKT | NWH | CBI | STA | MSQ | CMB | HMP | YRM | GUN | KJI | ERV | CLH | PTB | SYH | MBO |
| --- | --- | --- | --- | --- | --- | --- | --- | --- | --- | --- | --- | --- | --- | --- | --- |
| TKT | - | 0.013 | 0.433 | 0.000 | 0.000 | 0.000 | 0.000 | 0.000 | 0.000 | 0.000 | 0.000 | 0.000 | 0.000 | 0.000 | 0.000 |
| NWH | **0.010** | - | 0.048 | 0.000 | 0.000 | 0.000 | 0.000 | 0.000 | 0.000 | 0.000 | 0.000 | 0.000 | 0.000 | 0.000 | 0.000 |
| CBI | 0.001 | **0.008** | - | 0.001 | 0.000 | 0.000 | 0.000 | 0.000 | 0.000 | 0.000 | 0.000 | 0.000 | 0.000 | 0.000 | 0.000 |
| STA | **0.015** | **0.035** | **0.016** | - | 0.000 | 0.000 | 0.000 | 0.000 | 0.000 | 0.000 | 0.000 | 0.000 | 0.000 | 0.000 | 0.000 |
| MSQ | **0.059** | **0.083** | **0.065** | **0.026** | - | 0.002 | 0.100 | 0.000 | 0.000 | 0.000 | 0.000 | 0.000 | 0.000 | 0.000 | 0.000 |
| CMB | **0.085** | **0.115** | **0.095** | **0.042** | **0.007** | - | 0.000 | 0.000 | 0.000 | 0.000 | 0.000 | 0.000 | 0.000 | 0.000 | 0.000 |
| HMP | **0.039** | **0.062** | **0.044** | **0.011** | 0.002 | **0.011** | - | 0.000 | 0.000 | 0.000 | 0.000 | 0.000 | 0.000 | 0.000 | 0.000 |
| YRM | **0.181** | **0.220** | **0.196** | **0.118** | **0.045** | **0.022** | **0.061** | - | 0.005 | 0.000 | 0.048 | 0.000 | 0.000 | 0.000 | 0.000 |
| GUN | **0.171** | **0.204** | **0.187** | **0.109** | **0.042** | **0.020** | **0.056** | **0.005** | - | 0.000 | 0.000 | 0.000 | 0.000 | 0.000 | 0.000 |
| KJI | **0.254** | **0.293** | **0.270** | **0.179** | **0.088** | **0.055** | **0.111** | **0.008** | **0.015** | - | 0.188 | 0.000 | 0.000 | 0.000 | 0.002 |
| ERV | **0.222** | **0.259** | **0.237** | **0.153** | **0.068** | **0.043** | **0.089** | **0.003** | **0.008** | 0.001 | - | 0.000 | 0.000 | 0.000 | 0.000 |
| CLH | **0.325** | **0.365** | **0.340** | **0.240** | **0.137** | **0.099** | **0.165** | **0.031** | **0.047** | **0.009** | **0.019** | - | 0.000 | 0.000 | 0.489 |
| PTB | **0.357** | **0.399** | **0.372** | **0.266** | **0.163** | **0.123** | **0.190** | **0.050** | **0.070** | **0.023** | **0.039** | **0.006** | - | 0.002 | 0.080 |
| SYH | **0.388** | **0.434** | **0.403** | **0.293** | **0.194** | **0.155** | **0.219** | **0.081** | **0.104** | **0.049** | **0.067** | **0.023** | **0.012** | - | 0.001 |
| MBO | **0.298** | **0.342** | **0.314** | **0.222** | **0.127** | **0.092** | **0.150** | **0.031** | **0.045** | **0.011** | **0.023** | 0.000 | 0.005 | **0.018** | - |
| BRN | **0.317** | **0.360** | **0.332** | **0.231** | **0.134** | **0.100** | **0.161** | **0.037** | **0.058** | **0.019** | **0.031** | **0.007** | **0.012** | **0.030** | 0.001 |
| BDB | **0.309** | **0.353** | **0.323** | **0.231** | **0.134** | **0.101** | **0.159** | **0.038** | **0.055** | **0.016** | **0.028** | 0.002 | 0.004 | **0.020** | 0.001 |
| BCT | **0.288** | **0.331** | **0.304** | **0.209** | **0.117** | **0.080** | **0.141** | **0.026** | **0.040** | **0.008** | **0.018** | 0.000 | **0.006** | **0.021** | 0.001 |
| FTB | **0.083** | **0.112** | **0.091** | **0.049** | **0.018** | **0.020** | **0.021** | **0.049** | **0.042** | **0.085** | **0.070** | **0.132** | **0.157** | **0.185** | **0.118** |
| BTH | **0.092** | **0.123** | **0.103** | **0.053** | **0.016** | **0.009** | **0.021** | **0.030** | **0.030** | **0.064** | **0.051** | **0.108** | **0.131** | **0.164** | **0.099** |
| NOH | **0.110** | **0.140** | **0.116** | **0.066** | **0.019** | **0.011** | **0.027** | **0.023** | **0.027** | **0.052** | **0.043** | **0.089** | **0.109** | **0.141** | **0.084** |
| FRH | **0.235** | **0.274** | **0.247** | **0.166** | **0.081** | **0.053** | **0.105** | **0.019** | **0.030** | **0.018** | **0.024** | **0.026** | **0.041** | **0.065** | **0.029** |
| SGB | **0.360** | **0.405** | **0.375** | **0.272** | **0.172** | **0.134** | **0.197** | **0.063** | **0.089** | **0.036** | **0.052** | **0.015** | **0.008** | 0.002 | **0.013** |
| PTH | **0.388** | **0.430** | **0.403** | **0.288** | **0.182** | **0.140** | **0.211** | **0.064** | **0.083** | **0.032** | **0.049** | **0.008** | **0.005** | **0.010** | **0.012** |
| CNB | **0.374** | **0.419** | **0.389** | **0.278** | **0.177** | **0.136** | **0.204** | **0.064** | **0.088** | **0.035** | **0.052** | **0.009** | **0.008** | 0.006 | **0.017** |

**Table S4.** Pairwise *F*_ST_ for 25 sampling sites of European green crab (*Carcinus maenas*) shown below the diagonal with their corresponding significance (*P* values corrected for false discovery rate (FDR)) above the diagonal. All significant pairwise *F*_ST_ comparisons are indicated in bold after correction for FDR.

*Continued*

*Continued*

|  | BRN | BDB | BCT | FTB | BTH | NOH | FRH | SGB | PTH | CNB |
| --- | --- | --- | --- | --- | --- | --- | --- | --- | --- | --- |
| TKT | 0.000 | 0.000 | 0.000 | 0.000 | 0.000 | 0.000 | 0.000 | 0.000 | 0.000 | 0.000 |
| NWH | 0.000 | 0.000 | 0.000 | 0.000 | 0.000 | 0.000 | 0.000 | 0.000 | 0.000 | 0.000 |
| CBI | 0.000 | 0.000 | 0.000 | 0.000 | 0.000 | 0.000 | 0.000 | 0.000 | 0.000 | 0.000 |
| STA | 0.000 | 0.000 | 0.000 | 0.000 | 0.000 | 0.000 | 0.000 | 0.000 | 0.000 | 0.000 |
| MSQ | 0.000 | 0.000 | 0.000 | 0.000 | 0.000 | 0.000 | 0.000 | 0.000 | 0.000 | 0.000 |
| CMB | 0.000 | 0.000 | 0.000 | 0.000 | 0.000 | 0.000 | 0.000 | 0.000 | 0.000 | 0.000 |
| HMP | 0.000 | 0.000 | 0.000 | 0.000 | 0.000 | 0.000 | 0.000 | 0.000 | 0.000 | 0.000 |
| YRM | 0.000 | 0.000 | 0.000 | 0.000 | 0.000 | 0.000 | 0.000 | 0.000 | 0.000 | 0.000 |
| GUN | 0.000 | 0.000 | 0.000 | 0.000 | 0.000 | 0.000 | 0.000 | 0.000 | 0.000 | 0.000 |
| KJI | 0.000 | 0.000 | 0.002 | 0.000 | 0.000 | 0.000 | 0.000 | 0.000 | 0.000 | 0.000 |
| ERV | 0.000 | 0.000 | 0.000 | 0.000 | 0.000 | 0.000 | 0.000 | 0.000 | 0.000 | 0.000 |
| CLH | 0.023 | 0.233 | 0.403 | 0.000 | 0.000 | 0.000 | 0.000 | 0.000 | 0.000 | 0.000 |
| PTB | 0.002 | 0.101 | 0.015 | 0.000 | 0.000 | 0.000 | 0.000 | 0.015 | 0.006 | 0.003 |
| SYH | 0.000 | 0.001 | 0.000 | 0.000 | 0.000 | 0.000 | 0.000 | 0.343 | 0.004 | 0.093 |
| MBO | 0.377 | 0.354 | 0.368 | 0.000 | 0.000 | 0.000 | 0.000 | 0.006 | 0.001 | 0.000 |
| BRN | - | 0.206 | 0.001 | 0.000 | 0.000 | 0.000 | 0.000 | 0.011 | 0.000 | 0.000 |
| BDB | 0.004 | - | 0.330 | 0.000 | 0.000 | 0.000 | 0.000 | 0.033 | 0.001 | 0.000 |
| BCT | **0.012** | 0.001 | - | 0.000 | 0.000 | 0.000 | 0.000 | 0.000 | 0.000 | 0.001 |
| FTB | **0.129** | **0.129** | **0.110** | - | 0.000 | 0.000 | 0.000 | 0.000 | 0.000 | 0.000 |
| BTH | **0.106** | **0.108** | **0.087** | **0.015** | - | 0.221 | 0.000 | 0.000 | 0.000 | 0.000 |
| NOH | **0.090** | **0.092** | **0.073** | **0.016** | 0.001 | - | 0.000 | 0.000 | 0.000 | 0.000 |
| FRH | **0.027** | **0.030** | **0.020** | **0.075** | **0.051** | **0.039** | - | 0.000 | 0.000 | 0.000 |
| SGB | **0.016** | **0.009** | **0.016** | **0.164** | **0.139** | **0.117** | **0.048** | - | 0.011 | 0.296 |
| PTH | **0.018** | **0.012** | **0.012** | **0.172** | **0.149** | **0.124** | **0.041** | **0.009** | - | 0.113 |
| CNB | **0.022** | **0.017** | **0.013** | **0.169** | **0.141** | **0.119** | **0.044** | 0.002 | 0.003 | - |

**Table S5.** Sampling locations with latitude (lat) and longitude (long) for mitochondrial (COI) and microsatellite (micro) genetic markers for European green crab (*Carcinus maenus*) collected between 2000 and 2015. Sample number (N) and southern haplotype frequency (for COI) or coancestry coefficient (Q-value for microsatellites) are provided along with the estimated least cost distance of each site from Tuckerton, New Jersey (TKT). For COI data, model predictions for 2014 are provided and based on estimates from Pringle et al. (2011). Site codes are provide for samples collected in 2015 for reference in Figure S5.

| **Location name** | **Marker** | **Year** | **N** | **Southern haplotype frequency or Q-value** | **Distance from TKT (km)** | **Lat** | **Long** | **Site code for 2015** |
| --- | --- | --- | --- | --- | --- | --- | --- | --- |
| Freeport, NY | COI | 2000 | 17 | 1.00 | 128 | 40.62 | -73.58 |  |
| Nahant, MA | COI | 2000 | 7 | 1.00 | 550 | 42.38 | -70.96 |  |
| Winthrop, MA | COI | 2000 | 11 | 1.00 | 551 | 42.42 | -70.90 |  |
| Searsport, ME | COI | 2000 | 16 | 1.00 | 866 | 44.42 | -69.00 |  |
| Lubec, ME | COI | 2000 | 13 | 1.00 | 1047 | 44.82 | -66.95 |  |
| Chance Harbor, NB | COI | 2000 | 8 | 1.00 | 1112 | 45.12 | -66.35 |  |
| Scots Bay, NS | COI | 2000 | 18 | 0.83 | 1300 | 45.27 | -64.78 |  |
| Gullivers Cove, NS | COI | 2000 | 12 | 0.75 | 1418 | 44.49 | -66.09 |  |
| Grosses Coques, NS | COI | 2000 | 14 | 0.64 | 1434 | 44.35 | -66.11 |  |
| Cape Sable, NS | COI | 2000 | 14 | 0.21 | 1562 | 43.38 | -65.62 |  |
| Sandy Point/Gunning Cove, NS | COI | 2000 | 14 | 0.43 | 1615 | 43.68 | -65.23 |  |
| Broad Cove, NS* | COI | 2000 |  | 0.38 | 1682 | 44.17 | -64.47 |  |
| Chester, NS | COI | 2000 | 19 | 0.32 | 1734 | 44.53 | -64.23 |  |
| Murphy Cove, NS | COI | 2000 | 16 | 0.00 | 1754 | 44.47 | -63.77 |  |
| Halifax, NS | COI | 2000 | 14 | 0.14 | 1789 | 44.62 | -63.56 |  |
| Musquodoboit, NS | COI | 2000 | 10 | 0.10 | 1809 | 44.67 | -63.18 |  |
| Torbay, NS | COI | 2000 | 15 | 0.00 | 1971 | 45.18 | -61.35 |  |
| Guysborough, NS | COI | 2000 | 12 | 0.00 | 2052 | 45.38 | -61.48 |  |
| Port Hawkesbury, NS | COI | 2000 | 11 | 0.00 | 2052 | 45.53 | -61.29 |  |
| Rye, NY | COI | 2002 | 10 | 1.00 | 176 | 40.83 | -72.50 |  |
| Barnstable, MA | COI | 2002 | 20 | 1.00 | 451 | 41.70 | -70.28 |  |
| Nahant, MA | COI | 2002 | 24 | 1.00 | 550 | 42.42 | -70.90 |  |
| Isle of Shoals | COI | 2002 | 20 | 1.00 | 631 | 43.03 | -70.72 |  |
| Chebeague, ME | COI | 2002 | 19 | 1.00 | 719 | 43.73 | -70.10 |  |
| New Harbor, ME | COI | 2002 | 19 | 1.00 | 776 | 43.87 | -69.47 |  |
| Searsport, ME | COI | 2002 | 17 | 1.00 | 866 | 44.42 | -69.00 |  |
| Grand Manan Island, NB | COI | 2002 | 22 | 0.82 | 974 | 44.52 | -67.62 |  |
| Lubec, ME | COI | 2002 | 20 | 0.95 | 1047 | 44.83 | -66.98 |  |
| Chance Harbor, NB | COI | 2002 | 20 | 0.80 | 1112 | 45.12 | -66.35 |  |
| West Quaco, NB | COI | 2002 | 20 | 0.80 | 1196 | 45.35 | -65.54 |  |
| Cape Enrage, NB | COI | 2002 | 19 | 0.63 | 1258 | 45.59 | -64.79 |  |
| Cap D'Or, NS | COI | 2002 | 19 | 0.84 | 1274 | 45.80 | -64.38 |  |
| Scots Bay, NS | COI | 2002 | 19 | 1.00 | 1300 | 45.30 | -64.44 |  |
| Port George, NS | COI | 2002 | 20 | 0.90 | 1319 | 45.03 | -65.05 |  |
| Gullivers Cove, NS | COI | 2002 | 21 | 0.81 | 1418 | 44.49 | -66.09 |  |
| Grosses Coques, NS | COI | 2002 | 20 | 0.50 | 1434 | 44.35 | -66.10 |  |
| Cape Sable, NS | COI | 2002 | 19 | 0.37 | 1562 | 43.43 | -65.58 |  |
| Sandy Point / Gunning Cove, NS | COI | 2002 | 16 | 0.00 | 1615 | 43.67 | -65.00 |  |
| Broad Cove, NS* | COI | 2002 |  | 0.14 | 1682 | 44.17 | -64.47 |  |
| Chester, NS | COI | 2002 | 20 | 0.30 | 1734 | 44.53 | -64.23 |  |
| Murphy Cove, NS | COI | 2002 | 19 | 0.05 | 1754 | 44.47 | -63.77 |  |
| Halifax, NS | COI | 2002 | 20 | 0.25 | 1789 | 44.62 | -63.56 |  |
| Torbay, NS | COI | 2002 | 20 | 0.05 | 1971 | 45.18 | -61.35 |  |
| Guysborough, NS | COI | 2002 | 20 | 0.05 | 2052 | 45.55 | -61.28 |  |
| Port Hawkesbury, NS | COI | 2002 | 19 | 0.00 | 2052 | 45.55 | -61.28 |  |
| Antigonish, NS* | COI | 2002 |  | 0.00 | 2278 | 45.68 | -61.88 |  |
| Greenwich, CT | COI | 2007 | 13 | 0.85 | 183 | 41.05 | -72.47 |  |
| Barnstable, MA | COI | 2007 | 10 | 1.00 | 451 | 41.70 | -70.28 |  |
| Gloucester, MA | COI | 2007 | 20 | 0.85 | 576 | 42.61 | -70.65 |  |
| Rye / Odiorne, NH | COI | 2007 | 17 | 1.00 | 625 | 42.98 | -70.62 |  |
| Isle of Shoals | COI | 2007 | 20 | 1.00 | 631 | 43.03 | -70.72 |  |
| Falmouth, ME | COI | 2007 | 16 | 0.94 | 716 | 43.71 | -70.14 |  |
| Belfast, ME | COI | 2007 | 17 | 1.00 | 867 | 44.42 | -69.00 |  |
| Jonesport, ME | COI | 2007 | 16 | 0.88 | 970 | 44.47 | -67.57 |  |
| Lubec, ME | COI | 2007 | 7 | 0.43 | 1047 | 44.83 | -66.98 |  |
| Chance Harbor, NB | COI | 2007 | 14 | 0.57 | 1112 | 45.12 | -66.35 |  |
| West Quaco, NB | COI | 2007 | 16 | 0.56 | 1196 | 45.35 | -65.54 |  |
| Cap D'Or, NS | COI | 2007 | 25 | 0.76 | 1274 | 45.27 | -64.78 |  |
| Margarettsville, NS | COI | 2007 | 17 | 0.82 | 1313 | 45.16 | -64.36 |  |
| Kingsport, NS | COI | 2007 | 13 | 0.23 | 1330 | 45.00 | -65.15 |  |
| Gullivers Cove, NS | COI | 2007 | 19 | 0.58 | 1418 | 44.60 | -65.92 |  |
| Yarmouth, NS | COI | 2007 | 20 | 0.20 | 1498 | 43.80 | -66.15 |  |
| Cape Sable, NS | COI | 2007 | 15 | 0.07 | 1562 | 43.43 | -65.58 |  |
| Liverpool, NS | COI | 2007 | 15 | 0.87 | 1663 | 44.03 | -64.72 |  |
| Chester, NS | COI | 2007 | 17 | 0.41 | 1734 | 44.53 | -64.23 |  |
| Halifax, NS | COI | 2007 | 17 | 0.24 | 1789 | 44.62 | -63.56 |  |
| Tangier, NS | COI | 2007 | 17 | 0.00 | 1853 | 44.79 | -62.74 |  |
| Marie Joseph, NS | COI | 2007 | 17 | 0.00 | 1906 | 44.96 | -62.08 |  |
| Port Hawkesbury, NS | COI | 2007 | 18 | 0.11 | 2052 | 45.55 | -61.28 |  |
| Antigonish, NS* | COI | 2007 |  | 0.10 | 2278 | 45.76 | -62.65 |  |
| Goldsmith, NY (adult) | COI | 2015 | 23 | 1.00 | 244 | 41.00 | -73.57 | GLD |
| Barnstable, MA (adult) | COI | 2015 | 25 | 1.00 | 451 | 41.70 | -70.28 | BNS |
| Gloucester, MA | COI | 2015 | 12 | 1.00 | 576 | 42.60 | -70.67 | GLO |
| York, ME (adult/juvenile) | COI | 2015 | 23 | 1.00 | 640 | 43.15 | -70.62 | YOR |
| Portland, ME | COI | 2015 | 24 | 0.75 | 689 | 43.52 | -70.30 | POR |
| Boothbay Harbor, ME | COI | 2015 | 4 | 1.00 | 760 | 43.85 | -69.62 | BOT |
| Belfast, ME (adult/juvenile) | COI | 2015 | 36 | 0.92 | 867 | 44.45 | -68.91 | BEL |
| Bar Harbor, ME (adult/juvenile) | COI | 2015 | 61 | 0.80 | 911 | 44.38 | -68.20 | BAR |
| Roque Bluffs, ME (adult/juvenile) | COI | 2015 | 20 | 0.65 | 986 | 44.60 | -67.47 | ROQ |
| St. Andrews, NB | COI | 2015 | 19 | 0.84 | 1071 | 45.07 | -67.05 | STA |
| Musquash, NB | COI | 2015 | 18 | 0.44 | 1129 | 45.18 | -66.25 | MSQ |
| Cumberland Basin, NB | COI | 2015 | 20 | 0.35 | 1301 | 45.30 | -64.44 | CMB |
| Hampton, NS | COI | 2015 | 18 | 0.61 | 1339 | 44.91 | -65.35 | HMP |
| Yarmouth, NS | COI | 2015 | 15 | 0.20 | 1498 | 43.80 | -66.15 | YRM |
| Sandy Point / Gunning Cove, NS | COI | 2015 | 15 | 0.13 | 1615 | 43.67 | -65.00 | SP |
| Kejimkujik Park Seaside, NS | COI | 2015 | 13 | 0.08 | 1634 | 43.84 | -64.84 | KJI |
| East River Point, NS | COI | 2015 | 13 | 0.08 | 1739 | 44.57 | -64.15 | ERV |
| Cole Harbour, NS | COI | 2015 | 18 | 0.11 | 1792 | 44.63 | -63.41 | CLH |
| Port Bickerton, NS | COI | 2015 | 17 | 0.00 | 1942 | 45.09 | -61.73 | PTB |
| Port Harmon, NL | COI | 2015 | 9 | 0.00 | 2737 | 49.16 | -58.29 | PTH |
| North Harbour, NL | COI | 2015 | 15 | 0.53 | - | 47.86 | -54.10 | NOH |
| Boat Harbor, NL | COI | 2015 | 16 | 0.25 | - | 47.43 | -54.84 | BTH |
| Fair Haven, NL | COI | 2015 | 7 | 0.43 | - | 47.54 | -53.89 | FRH |
| Fortune Bay, NL | COI | 2015 | 12 | 0.58 | - | 47.59 | -54.86 | FTB |
| Goldsmith, NY | COI | 2014 prediction |  | 0.96 | 244 | 41.00 | -73.57 |  |
| Barnstable, MA | COI | 2014 prediction |  | 0.92 | 451 | 41.70 | -70.28 |  |
| Gloucester, MA | COI | 2014 prediction |  | 0.90 | 576 | 42.60 | -70.67 |  |
| York, ME | COI | 2014 prediction |  | 0.82 | 640 | 43.15 | -70.62 |  |
| Portland, ME | COI | 2014 prediction |  | 0.70 | 689 | 43.52 | -70.30 |  |
| Belfast, ME | COI | 2014 prediction |  | 0.60 | 867 | 44.45 | -68.91 |  |
| Bar Harbor, ME | COI | 2014 prediction |  | 0.50 | 911 | 44.38 | -68.20 |  |
| Roque Bluffs, ME | COI | 2014 prediction |  | 0.45 | 986 | 44.60 | -67.47 |  |
| St. Andrews, NB | COI | 2014 prediction |  | 0.35 | 1071 | 45.07 | -67.05 |  |
| Musquash, NB | COI | 2014 prediction |  | 0.35 | 1129 | 45.18 | -66.25 |  |
| Cumberland Basin, NB | COI | 2014 prediction |  | 0.30 | 1301 | 45.30 | -64.44 |  |
| Hampton, NS | COI | 2014 prediction |  | 0.30 | 1339 | 44.91 | -65.35 |  |
| Yarmouth, NS | COI | 2014 prediction |  | 0.30 | 1498 | 43.80 | -66.15 |  |
| Sandy Point / Gunning Cove, NS | COI | 2014 prediction |  | 0.25 | 1615 | 43.67 | -65.00 |  |
| Kejimkujik Park Seaside, NS | COI | 2014 prediction |  | 0.25 | 1634 | 43.84 | -64.84 |  |
| East River Point, NS | COI | 2014 prediction |  | 0.20 | 1739 | 44.57 | -64.15 |  |
| Cole Harbour, NS | COI | 2014 prediction |  | 0.15 | 1792 | 44.63 | -63.41 |  |
| Port Bickerton, NS | COI | 2014 prediction |  | 0.10 | 1942 | 45.09 | -61.73 |  |
| Moosepoint, ME | micro | 2000 | 15 | 0.95 | 868 | 44.45 | -68.92 |  |
| Lubec, ME | micro | 2000 | 5 | 0.91 | 1043 | 44.82 | -66.95 |  |
| Chance Harbour, NB | micro | 2000 | 4 | 0.79 | 1112 | 45.12 | -66.35 |  |
| Gulliver's Cove, NS | micro | 2000 | 9 | 0.94 | 1397 | 44.49 | -66.09 |  |
| Grosses Coques, NS | micro | 2000 | 8 | 0.43 | 1434 | 44.35 | -66.10 |  |
| Cape Sable, NS | micro | 2000 | 12 | 0.41 | 1561 | 43.38 | -65.62 |  |
| Sandy Point, NS | micro | 2000 | 12 | 0.79 | 1598 | 43.67 | -65.00 |  |
| Broad Cove, NS | micro | 2000 | 7 | 0.52 | 1682 | 44.17 | -64.47 |  |
| Chester, NS | micro | 2000 | 9 | 0.39 | 1734 | 44.53 | -64.23 |  |
| Halifax, NS | micro | 2000 | 12 | 0.23 | 1789 | 44.62 | -63.56 |  |
| Murphy's Cove, NS | micro | 2000 | 9 | 0.10 | 1851 | 44.74 | -62.64 |  |
| Tor Bay, NS | micro | 2000 | 10 | 0.10 | 1971 | 45.18 | -61.35 |  |
| Louisborg NS | micro | 2000 | 10 | 0.05 | 2138 | 45.92 | -59.95 |  |
| Rye Playland, NY | micro | 2002 | 9 | 0.97 | 177 | 41.05 | -72.47 |  |
| Nahant, MA | micro | 2002 | 21 | 0.98 | 550 | 42.42 | -70.90 |  |
| Isles of Shoals, NH | micro | 2002 | 19 | 0.98 | 630 | 42.99 | -70.61 |  |
| Falmouth, ME | micro | 2002 | 18 | 0.98 | 716 | 43.72 | -70.23 |  |
| Chance Harbour, NB | micro | 2002 | 20 | 0.88 | 1112 | 45.13 | -66.35 |  |
| Gullivers Cove, NS | micro | 2002 | 20 | 0.84 | 1397 | 44.49 | -66.09 |  |
| Cape Sable, NS | micro | 2002 | 19 | 0.25 | 1562 | 43.44 | -65.58 |  |
| Sandy Point, NS | micro | 2002 | 15 | 0.13 | 1598 | 43.67 | -65.00 |  |
| Broad Cove, NS | micro | 2002 | 21 | 0.14 | 1682 | 44.17 | -64.47 |  |
| Chester, NS | micro | 2002 | 19 | 0.38 | 1734 | 44.53 | -64.23 |  |
| Halifax, NS | micro | 2002 | 20 | 0.15 | 1789 | 44.62 | -63.56 |  |
| Murphys Cove, NS | micro | 2002 | 21 | 0.05 | 1851 | 44.78 | -62.75 |  |
| St Peters, NS | micro | 2002 | 20 | 0.06 | 2065 | 45.65 | -60.87 |  |
| Rye Playland, NY | micro | 2007 | 12 | 0.96 | 187 | 40.95 | -73.67 |  |
| Nahant, MA | micro | 2007 | 19 | 0.94 | 580 | 42.60 | -70.67 |  |
| Odiorne, NH | micro | 2007 | 17 | 0.97 | 623 | 42.98 | -70.62 |  |
| Isles of Shoals, NH | micro | 2007 | 20 | 0.93 | 630 | 43.01 | -70.74 |  |
| Falmouth, ME | micro | 2007 | 16 | 0.94 | 715 | 43.70 | -70.22 |  |
| Lubec, ME | micro | 2007 | 8 | 0.91 | 1043 | 44.82 | -66.95 |  |
| Gulliver's Cove, NS | micro | 2007 | 20 | 0.44 | 1418 | 44.61 | -65.92 |  |
| Cape Sable, NS | micro | 2007 | 15 | 0.25 | 1561 | 43.38 | -65.62 |  |
| Halifax, NS | micro | 2007 | 21 | 0.16 | 1789 | 44.62 | -63.56 |  |
| Marie Joseph, NS | micro | 2007 | 20 | 0.06 | 1906 | 44.96 | -62.07 |  |
| St Peters, NS | micro | 2007 | 23 | 0.07 | 2065 | 45.65 | -60.87 |  |
| Goldsmith's Inlet, NY | micro | 2015 | 27 | 0.93 | 244 | 41.00 | -73.62 | GLD |
| Martha's Vineyard, MA | micro | 2015 | 30 | 0.90 | 451 | 41.70 | -70.28 | CCMV |
| York, ME | micro | 2015 | 27 | 0.88 | 640 | 43.15 | -70.62 | YOR |
| Portland, ME | micro | 2015 | 37 | 0.91 | 691 | 43.52 | -70.30 | POR |
| Camden/Stockton, ME | micro | 2015 | 50 | 0.84 | 863 | 44.39 | -68.98 | CAMSTK |
| Bar Harbor, ME | micro | 2015 | 49 | 0.83 | 913 | 44.38 | -68.20 | BAR |
| Musquash, NB | micro | 2015 | 50 | 0.60 | 1129 | 45.18 | -66.25 | MSQ |
| Gunning Cove, NS | micro | 2015 | 40 | 0.20 | 1614 | 43.68 | -65.24 | GUN |
| Cole Harbor, NS | micro | 2015 | 50 | 0.06 | 1792 | 44.65 | -63.43 | CLH |
| Port Bickerton, NS | micro | 2015 | 50 | 0.05 | 1941 | 45.11 | -61.73 | PTB |

*No sample size because the sample sizes were based on those provided by Pringle et al. (2011)

## Supplemental figures


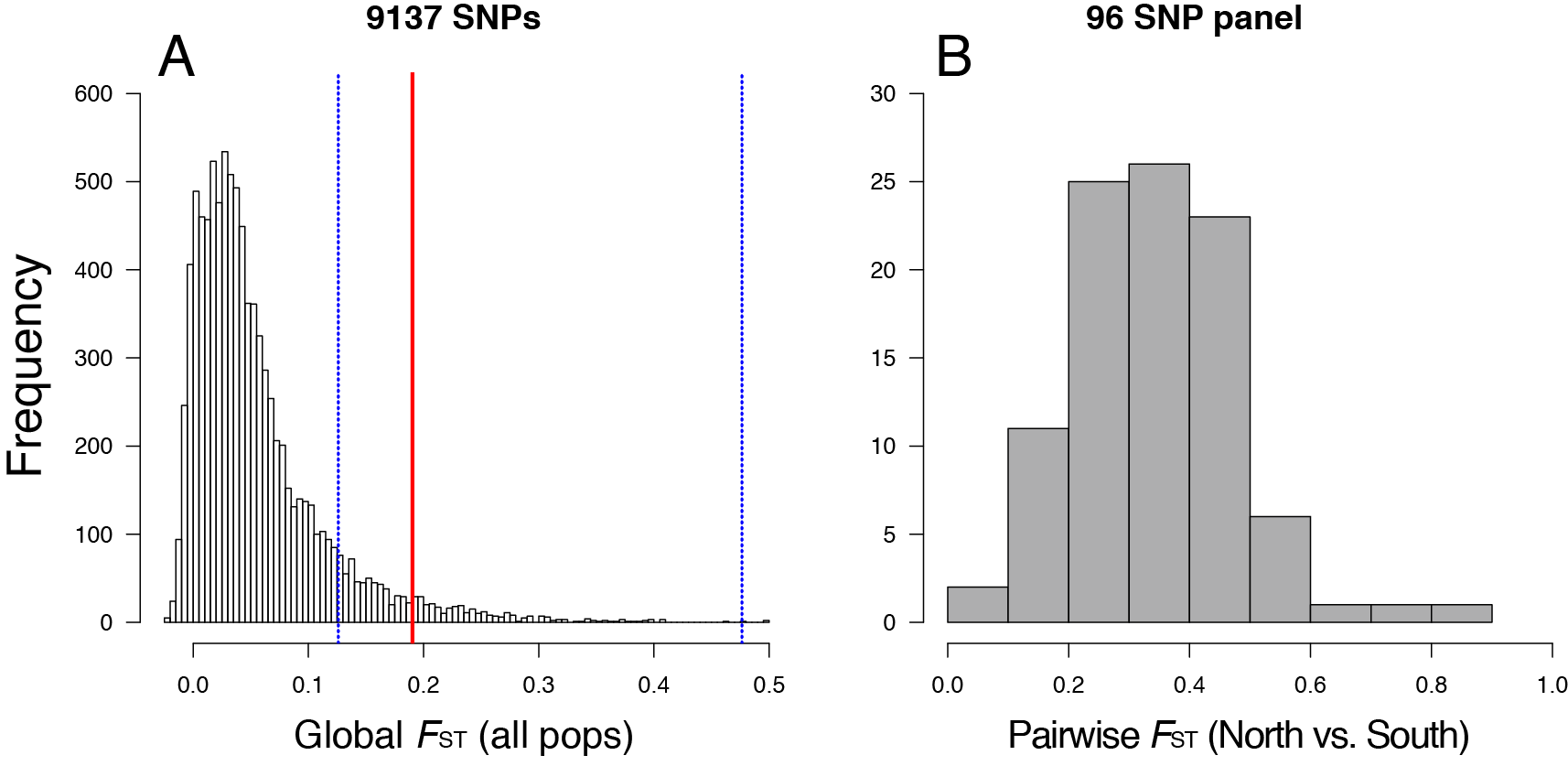


**Figure S1.** **(A)** Histogram showing distribution of locus-specific global *F*_ST_ values calculated across 11 populations of *Carcinus maenas* in eastern North America from Jeffery et al. (2017). A panel of 96 loci with high-*F*_ST_ and low linkage disequilibrium was chosen from this distribution and the panel mean and range *F*_ST_ across the 11 populations are indicated by the solid red and dashed blue lines, respectively. The diagnostic power of the panel is shown in **(B)** a histogram with the distribution of locus-specific pairwise *F*_ST_ values between the southernmost site (Tuckerton, New Jersey) and northernmost site (Corner Brook, Newfoundland).


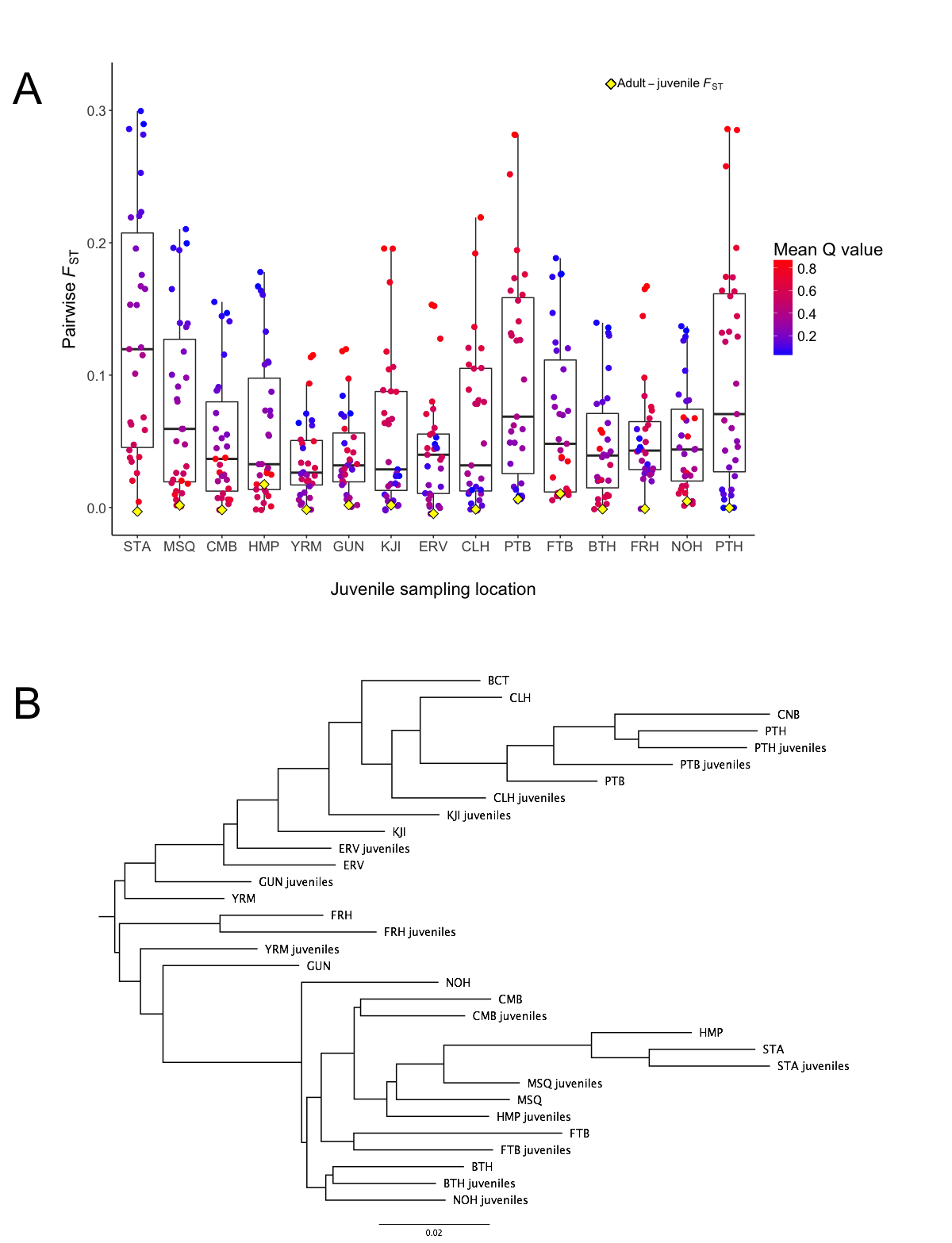


**Figure S2.** **(A)** Pairwise *F*_ST_ for all juvenile sampling sites of European green crab (*Carcinus maenas*), where *F*_ST_ was calculated against all 2015 adult and juvenile locations (31 pairwise comparisons per juvenile site). *F*_ST_ values are coloured based on mean Q-values determined by the program STRUCTURE. Yellow diamonds indicate the pairwise *F*_ST_ value between adult and juveniles collected at the same location. **(B)** Neighbour-joining tree of all 2015 adult and juvenile *C. maenas* sampling locations based on Cavalli-Sforza & Edwards (1967) chord distances for 96 SNPs.

**
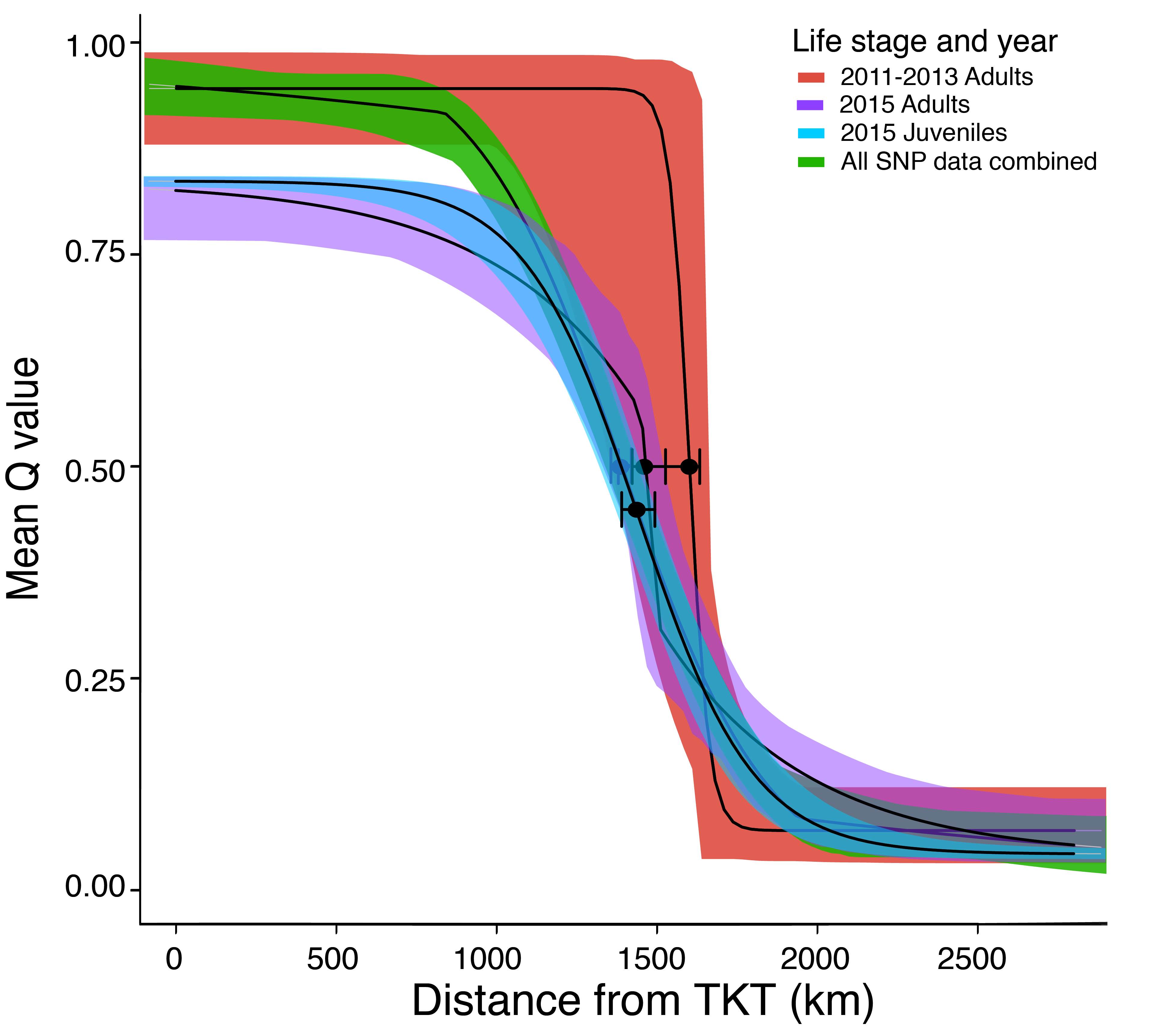
**

**Figure S3.** Maximum likelihood (ML) genetic clines for European green crab (*Carcinus maenas*) for different life stages and years where clines were modelled using *hzar* (Derryberry et al., 2014) with mean admixture coefficient against the distance from Tuckerton, New Jersey (TKT) for each site. Q-values were determined in STRUCTURE using a panel of 96 diagnostic SNPs. Clines are shown with their associated fuzzy cline region (95% credible cline region) and the ML estimate of cline centre is indicated (dot) with two log-likelihood low and high estimates (whiskers). We note that only one site was sampled in 2013 and thus was included with the 2011 samples, and sites in southern Newfoundland were excluded from the analyses. Models were weighted based on effective number of alleles in the population (see Methods).


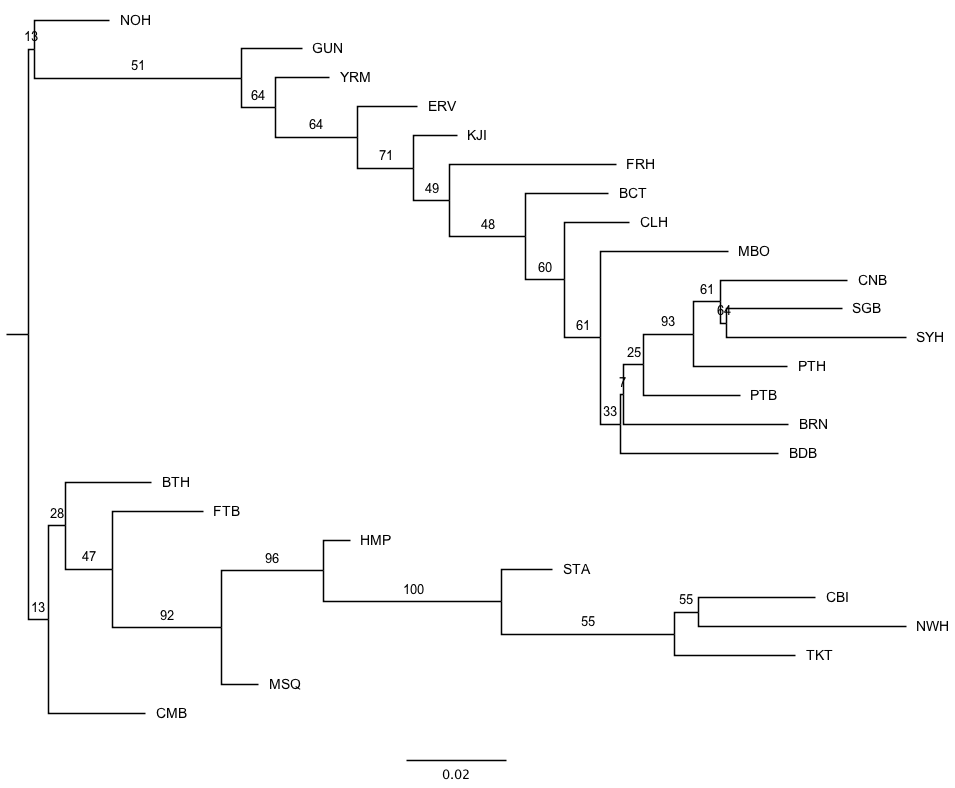


**Figure S4.** Neighbour-joining tree of 25 European green crab (*Carcinus maenas*) sampling locations based on Cavalli-Sforza & Edwards (1967) chord distances for 96 SNPs. Bootstrap values from 1000 bootstrap pseudoreplicates are present at nodes.


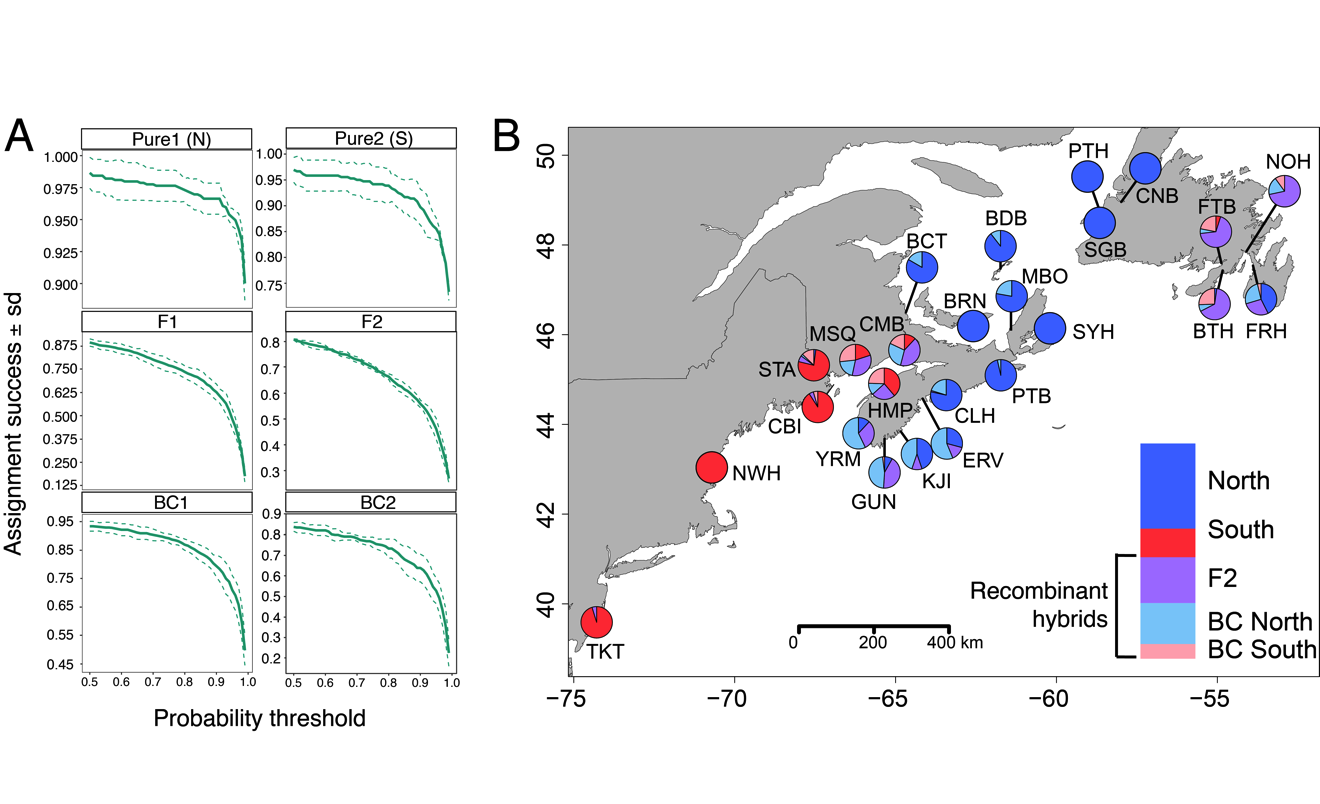


**Figure S5. (A)** Mean (± standard error) assignment success from nine simulated datasets (three simulated datasets with three replicates each) of European green crab (*Carcinus maenas*) for six genotype classes including pure north, pure south, F_1_ hybrids, F_2_ hybrids, and both backcrosses using the program NEWHYBRIDS (Anderson, 2008) and *hybriddetective* (Wringe et al., 2017). **(B)** Map of 25 sampling locations with the proportion of individuals assigned to pure or hybrid genotype classes. Inset bar plots show the proportion of individuals assigned to each genotype class across all sites. No F_1_ hybrids were detected in the study range and all second-generation hybrids (F_2_ and backcrosses) were combined and considered recombinant hybrids in our study.


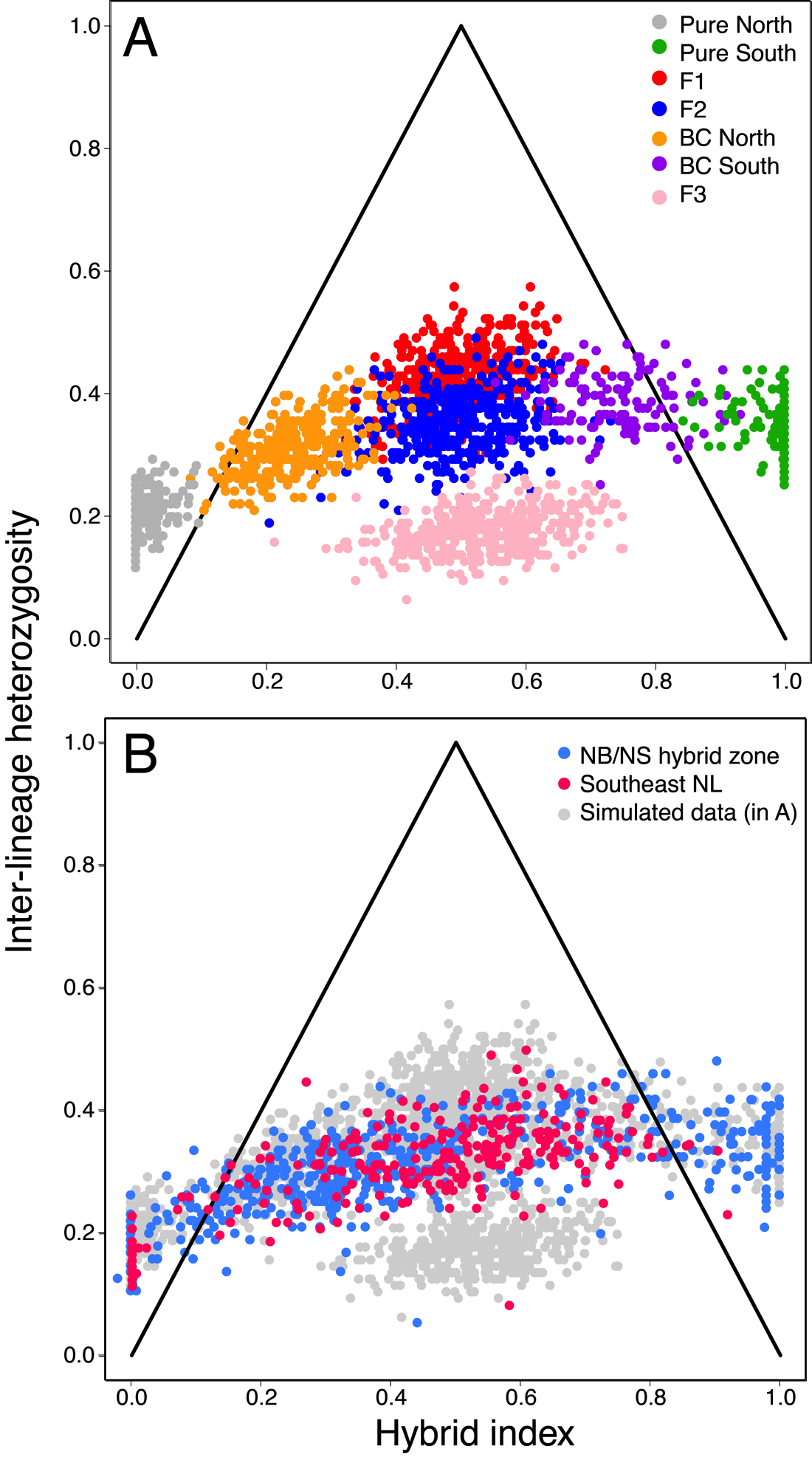


**Figure S6.** Triangle plots generated using *INTROGRESS* (Gompert & Buerkle, 2010) where maximum likelihood hybrid index is plotted against inter-lineage heterozygosity. Panel **(A)** shows results for a simulated multigenerational hybrid dataset and **(B)** shows results for the individuals from the hybrid zone (blue) and the hybrid region of southeastern Newfoundland (red) with simulated (gray) data from panel (A).


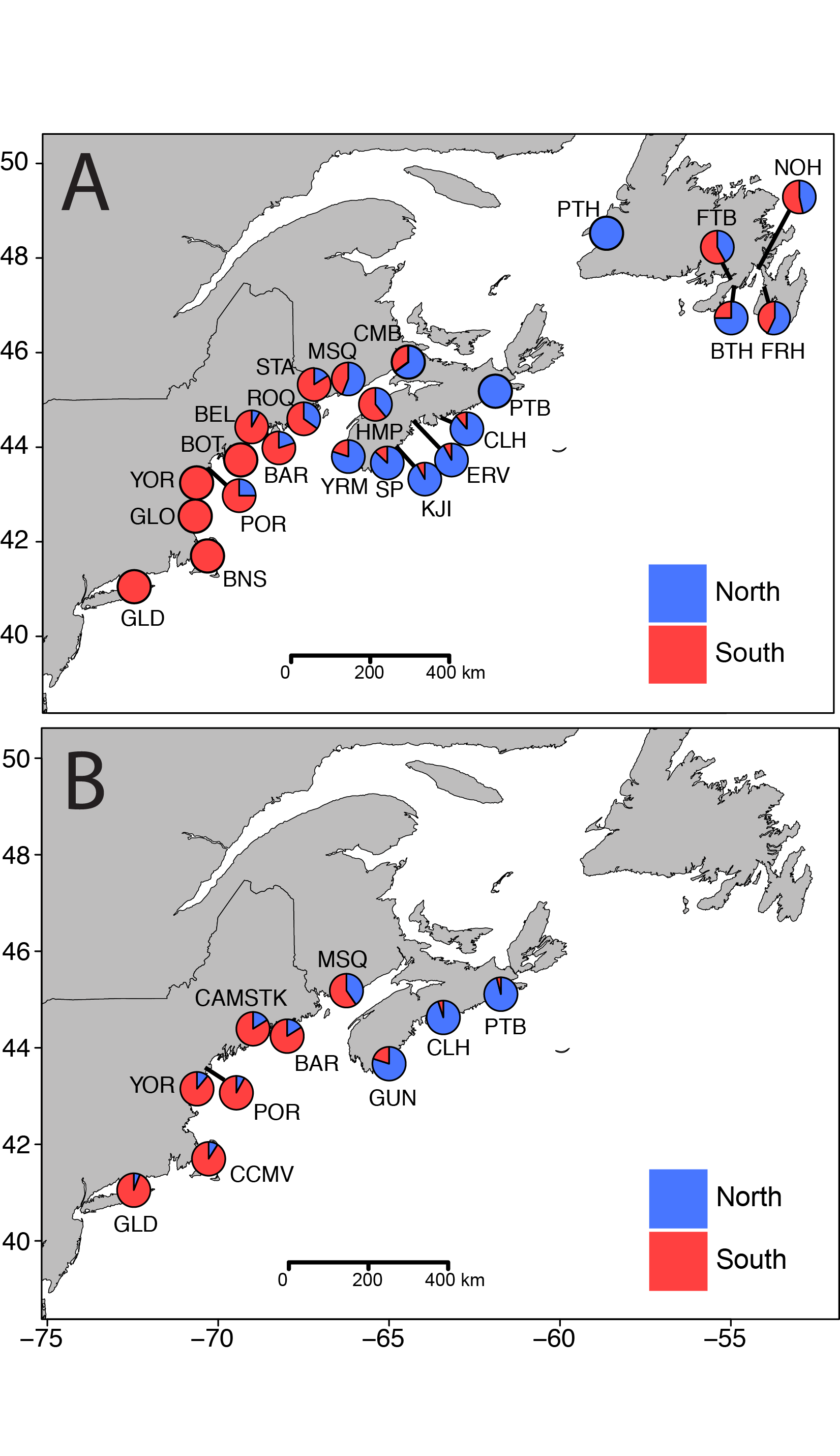


**Figure S7.** Map of population structure for **(A)** the mitochondrial cytochrome *c* oxidase subunit I (COI) gene and **(B)** microsatellite markers for European green crab (*Carcinus maenas*) young-of-the-year collected in 2015. Panel **(A)** shows northern (blue) and southern (red) mitochondrial haplotype frequencies and panel **(B)** shows the results of Bayesian clustering analysis using 11 microsatellite loci with the proportion of membership assigned to two genetic clusters (north and south) for each site. Site codes are provided in Table S5.


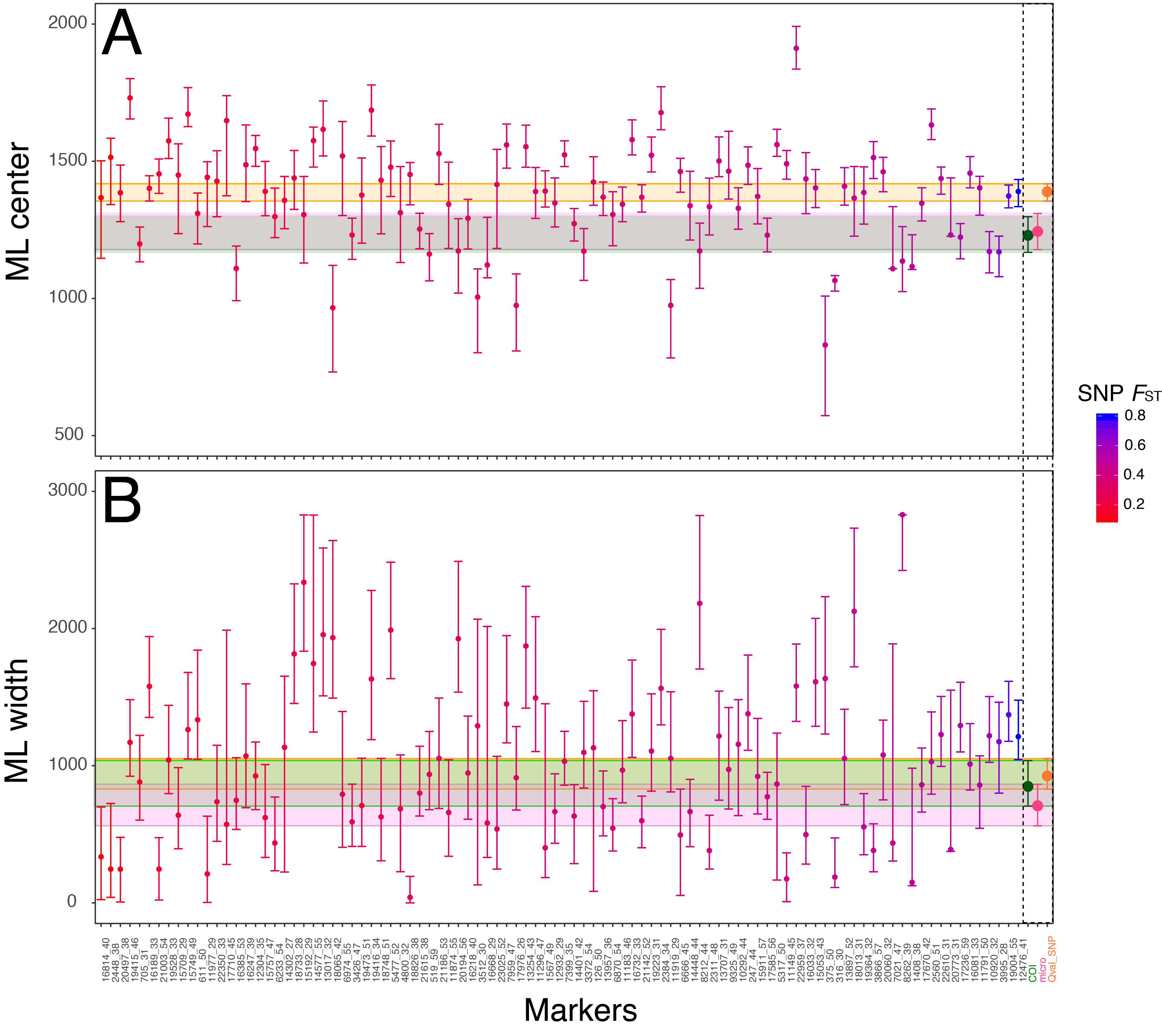

**Figure S8.** Maximum likelihood (ML) cline (A) centres and (B) widths in kilometres for all 96 SNP markers arranged from lowest to highest *F*_ST_ where *F*_ST_ represents pairwise genetic divergence between northernmost and southernmost sites (see Fig. S1). Error bars represent two log likelihood support limits for cline estimates. ML cline centre and widths are provided (and separated by a dashed line) for 2015 COI cline (green), 2015 microsatellite cline (pink) and SNP Q-value cline (orange). Coloured horizontal bars indicate support limits for ML cline estimates for COI, microsatellite and Q-value clines.

## Literature cited

Anderson, E. C. (2003). User’s guide to the program NEWHYBRIDS version 1.1 beta. Technical Report, 7 April 2003.

Anderson, E. C. (2008). Bayesian inference of species hybrids using multilocus dominant genetic markers. *Philosophical Transactions of the Royal Society of London B*, *363*, 2841-2850.

Blakeslee, A. M. H., McKenzie, C. H., Darling, J. A., Byers, J. E., Pringle, J. M., & Roman, J. (2010). A hitchhiker’s guide to the Maritimes: anthropogenic transport facilitates long-distance dispersal of an invasive marine crab to Newfoundland. *Diversity and Distributions*, *16*, 879-891.

Buerkle, C. A. (2005). Maximum-likelihood estimation of a hybrid index based on molecular markers. *Molecular Ecology Notes*, *5*, 684-687.

Cavalli-Sforza, L. L., & Edwards, A. W. F. (1967). Phylogenetic analysis models and estimation procedures. *American Journal of Human Genetics*, *19*, 233-257.

Clement, M., Posada, D., & Crandall, K. A. (2000). TCS: a computer program to estimate gene genealogies. *Molecular Ecology*, *9*, 1657-1659.

Darling, J. A., Bagley, M. J., Roman, J., Tepolt, C. K., & Geller, J. B. (2008). Genetic patterns across multiple introductions of the globally invasive crab genus *Carcinus*. *Molecular Ecology*, *17*, 4992-5007.

Darling, J. A., Tsai, Y. H., Blakeslee, A. M., & Roman, J. (2014). Are genes faster than crabs? Mitochondrial introgression exceeds larval dispersal during population expansion of the invasive crab *Carcinus maenas*. *Royal Society Open Science*, *1*, 140202.

Derryberry, E. P., Derryberry, G. E., Maley, J. M., & Brumfield, R. T. (2014). HZAR: hybrid zone analysis using an R software package. *Molecular Ecology Resources*, *14*, 652-63.

Evanno, G., Regnaut, S., & Goudet, J. (2005). Detecting the number of clusters of individuals using the software STRUCTURE: a simulation study. *Molecular Ecology*, *14*, 2611-2620.

France, S. C., Rosel, P. E., Agenbroad, J. E., Mullineaux, L. S., & Kocher, T. D. (1996). DNA sequence variation of mitochondrial large-subunit rRNA provides support for a two-subclass organization of the Anthozoa (Cnidaria). *Molecular Marine Biology and Biotechnology*, *5*, 15-28.

Gompert, Z., & Buerkle, A. C. (2010). INTROGRESS: a software package for mapping components of isolation in hybrids. *Molecular Ecology Resources*, *10*, 378-384.

Jeffery, N. W., DiBacco, C., Van Wyngaarden, M., Hamilton, L. C., Stanley, R. R. E., Bernier, R., ... Bradbury, I. R. (2017). RAD sequencing reveals genomewide divergence between independent invasions of the European green crab (*Carcinus maenas*) in the Northwest Atlantic. *Ecology and Evolution*, *7*, 2513-2524.

Jeffery, N. W., DiBacco, C., Wringe, B. F., Stanley, R. R. E., Hamilton, L. C., Ravindran, P. N., & Bradbury, I. R. (2017a). Genomic evidence of hybridization between two independent invasions of European green crab (*Carcinus maenas*) in the Northwest Atlantic. *Heredity*, *119*, 154–165.

Keenan, K., McGinnity, P., Cross, T. F., Crozier, W. W., Prodöhl, P. A., & O'Hara, R. B. (2013). diveRsity: An R package for the estimation and exploration of population genetics parameters and their associated errors. *Methods in Ecology and Evolution*, *4*, 782-788.

Larkin, M. A., Blackshields, G., Brown, N. P., Chenna, R., McGettigan, P. A., McWilliam, H., ... Higgins, D. G. (2007). Clustal W and clustal X version 2.0. *Bioinformatics*, *23*, 2947-2948.

Macholán, M., Baird, S. J., Munclinger, P., Dufková, P., Bímová, B., & Piálek, J. (2008). Genetic conflict outweighs heterogametic incompatibility in the mouse hybrid zone? *BMC Evolutionary Biology*, *8*, 271.

Meirmans, P. G., & Van Tienderen, P. H. (2004). genotype and genodive: two programs for the analysis of genetic diversity of asexual organisms. *Molecular Ecology Notes*, *4*, 792-794.

Pascoal, S., Creer, S., Taylor, M. I., Queiroga, H., Carvalho, G., & Mendo, S. (2009). Development and application of microsatellites in *Carcinus maenas*: genetic differentiation between northern and central Portuguese populations. *PLoS One*, *4*, e7268.

Pringle, J. M., Blakeslee, A. M., Byers, J. E., & Roman, J. (2011). Asymmetric dispersal allows an upstream region to control population structure throughout a species' range. *Proceedings of the National Academy of Sciences*, *108*, 15288-15293.

Pritchard, J. K., Stephens, M., & Donnelly, P. (2000). Inference of population structure using multilocus genotype data. *Genetics*, *155*, 945-959.

Roman, J. (2006). Diluting the founder effect: cryptic invasions expand a marine invader's range. *Proceedings of the Royal Society of London B: Biological Sciences*, *273*, 2453-2459.

Roman, J., & Palumbi, S. R. (2004). A global invader at home: population structure of the green crab, *Carcinus maenas*, in Europe. *Molecular Ecology*, *13*, 2891-2898.

Tepolt, C. K., Bagley, M. J., Geller, J. B., & Blum, M. J. (2006). Characterization of microsatellite loci in the European green crab (*Carcinus maenas*). *Molecular Ecology Notes*, *6*, 343-345.

Wringe, B. F., Stanley, R. R., Jeffery, N. W., Anderson, E. C., & Bradbury, I. R. (2017). *hybriddetective*: a workflow and package to facilitate the detection of hybridization using genomic data in R. *Molecular Ecology Resources*, *17*, e275–e284.

Wringe, B. F., Stanley, R. R., Jeffery, N. W., Anderson, E. C., & Bradbury, I. R. (2017). parallelnewhybrid: an R package for the parallelization of hybrid detection using newhybrids. *Molecular Ecology Resources*, *17*, 91-95.
